# Late-onset GM2 gangliosidosis: magnetic resonance imaging, diffusion tensor imaging, and correlational fiber tractography differentiate Tay–Sachs and Sandhoff diseases

**DOI:** 10.1007/s00415-025-13091-3

**Published:** 2025-04-23

**Authors:** Connor J. Lewis, Selby I. Chipman, Jean M. Johnston, Maria T. Acosta, Camilo Toro, Cynthia J. Tifft

**Affiliations:** Office of the Clinical Director and Medical Genetics Branch, National Human Genome Research Institute, National Institutes of Health, 10 Center Drive, Bethesda, MD USA

**Keywords:** GM2 gangliosidosis, Late-onset Tay–Sachs, Late-onset Sandhoff, Correlational tractography, Neuroimaging biomarkers

## Abstract

GM2 gangliosidosis is lysosomal storage disorder caused by deficiency of the heterodimeric enzyme β-hexosaminidase A. Tay–Sachs disease is caused by variants in *HEXA* encoding the α-subunit and Sandhoff disease is caused by variants in *HEXB* encoding the β-subunit. Due to shared clinical and biochemical findings, the two have been considered indistinguishable. We applied T1-weighted volumetric analysis, diffusion tensor imaging (DTI), and correlational fiber tractography to assess phenotypic differences in these two diseases. 51 T1-weighted and 40 DTI scans from 19 Late-Onset GM2 patients with either late-onset Sandhoff disease (LOSD), or late-onset Tay–Sachs (LOTS) were included and compared to 1033 neurotypical control volumetric MRI scans. LOTS patients had significantly smaller cerebellum volume compared to neurotypical controls (*p* < 0.0001) and LOSD patients (*p* < 0.0001). There was no statistical difference for the volume of any structure between LOSD and neurotypical controls. DTI analysis showed LOTS patients had higher mean diffusivity (MD) in the left cerebellum (*p* = 0.003703), right cerebellum (*p* = 0.003435), superior cerebellar peduncle (*p* = 0.007332), and vermis (*p* = 0.01007) compared to LOSD. LOTS patients had lower fractional anisotropy (FA) in the left cerebellum (*p* = 0.005537), right cerebellum (*p* = 0.01905), SCP (*p* = 0.02844), and vermis (*p* = 0.02469) when compared to LOSD. Correlational fiber tractography identified fiber tracts in cerebellar pathways with higher FA and lower MD in LOSD patients compared to LOTS patients. Our study shows neurobiologic differences between these two related disorders. To our knowledge, this is the first study using correlational tractography in a lysosomal storage disorder. This result indicates a greater burden of cerebellar pathology in LOTS patients compared with LOSD patients.

**Supplementary Information:**

The online version contains supplementary material available at 10.1007/s00415-025-13091-3.

## Introduction

GM2 gangliosidoses are autosomal recessive neurodegenerative lysosomal storage disorders caused by the cytotoxic accumulation of GM2 ganglioside. β-hexosaminidase A is the deficient enzyme and is responsible for degrading GM2 into GM3 as a part of the ganglioside catabolism pathway [1, 2]. Three diseases are associated with GM2 gangliosidosis, Tay–Sachs disease is characterized by biallelic variants in *HEXA* encoding the α subunit of β-hexosaminidase A. Sandhoff disease is characterized by biallelic variants in *HEXB* encoding the ß subunit resulting in deficiencies in both hexosaminidase A and B. GM2 activator deficiency, known as the AB variant, is characterized by biallelic variants in *GM2A* [3]. The resulting accumulation of GM2 gangliosides is toxic primarily to neurons where gangliosides play a key role in central nervous system function.

The frequency of Tay–Sachs disease is estimated between 1 in 200,000 and 1 in 320,000 individuals [4, 5]. The estimated prevalence for Sandhoff disease is between 1 in 500,000 and 1 in 1,500,000 individuals [6]. The AB activator deficiency is the rarest form of GM2 gangliosidosis with only 13 known cases reported [7]. There are no approved therapies for GM2 gangliosidosis, however investigations into enzyme replacement therapy, substrate reduction therapy, and gene therapy as potential treatments are currently underway [8–10].

Sandhoff and Tay–Sachs diseases can be further classified into three subtypes based on symptom onset age and disease progression corresponding to residual enzyme activity [6, 11]. The infantile classifications of these diseases are the most severe with symptom onset before 6 months and death by 5 years of age [12, 13]. The juvenile form of these diseases is less severe with symptom onset between 2 and 6 years and death within the second decade [12–14]. Adult or late onset GM2 gangliosidosis is the least severe form of the disease with symptom onset frequently occurring between adolescence and early adulthood and life expectancy reduced compared to unaffected adults [11, 15, 16]. The adult or late-onset form of GM2 gangliosidosis is also differentiated from the juvenile form due to preserved cognitive function [11, 12]. While the infantile and juvenile forms of the activator deficiency have been described, an adult-onset form of the activator deficiency has not been described to the best of our knowledge [7, 17, 18].

Due to similarities in clinical presentations and shared β-hexosaminidase A enzyme deficiency, late-onset Sandhoff (LOSD) and Tay–Sachs (LOTS) have been presumed indistinguishable. Patients with late-onset GM2 gangliosidosis typically present with a combination of neurogenic lower extremity weakness and coordination difficulties causing frequent falls [19, 20]. Dysarthria, oculomotor abnormalities, and tremors have also been frequently documented early in the disease course [19]. Neuropsychiatric symptoms in early adulthood have also been documented in late-onset GM2 gangliosidosis [19, 20]. Compared to the infantile and juvenile versions of GM2 gangliosidosis, late-onset GM2 gangliosidosis has the most variability in terms of symptoms spectrum and disease progression [19].

Recent studies have focused on differentiating these two forms of GM2 gangliosidosis [16, 21, 22]. Most often, both disorders lead to lower limb weakness disproportionally affecting the hip flexors and knee extensor muscles. Upper limb weakness, most prominent in the triceps, appears later in the disease course [19]. A slightly lower age of symptom onset, higher prevalence of psychosis, and dysarthria appear more commonly associated with LOTS, whereas length dependent sensory peripheral neuropathy including burning pain in feet and hands and dysautonomia are more typical for LOSD patients [16, 22, 23].

Case studies of MRI findings in late onset GM2 gangliosidosis at different stages of the disease have demonstrated numerous findings affecting the thalamus, enlargement of the 4th ventricle [24, 25], white matter abnormalities, atrophy of the cerebral cortex [22], brainstem [22], corpus callosum [22], and cerebellar atrophy (including gray and white matter) [26–28]. Previous studies have also mentioned cerebellar atrophy and enlarged 4th ventricle which are more prevalent and severe in LOTS disease as opposed to LOSD disease [16, 22, 23, 26]. Magnetic resonance spectroscopy (MRS), an imaging technique analyzing metabolite concentration in vivo has shown pathogenic differences associated with GM2 gangliosidosis [27]. Furthermore, MRS has highlighted cerebellar metabolic differences between LOTS and LOSD disease patients [23].

Diffusion weighted imaging (DWI) is a noninvasive neuroimaging modality with the capability of analyzing white matter microstructure based on the relative diffusion and diffusion restriction in the brain [29]. A previous study in the GM2 mouse model of Sandhoff disease has demonstrated a reduced apparent diffusion coefficient compared with wild type mice in the cortex, striatum, and thalamus [30]. However, we were unable to find any studies in humans.

Diffusion tensor imaging (DTI) builds on DWI by evaluating the diffusion tensor matrix and allows for the calculation of fiber tractography [31, 32]. DTI metrics include fractional anisotropy (FA) which is useful in characterizing white matter integrity, mean diffusivity (MD), axial diffusivity (AD), and radial diffusivity (RD). AD is a measure of the diffusion along the principal axis or parallel along an axon where decreased AD has been associated with axonal injury [33]. AD has been shown to increase with structural brain changes that accompany aging, Huntington’s disease and Alzheimer’s disease [34–37]. FA is a measure of directional diffusion restriction and pathogenic reductions in FA may represent reduced axonal packing, integrity [33]. MD is an average of the diffusion and pathogenic increases in MD may represent reduced white matter integrity [33]. RD is a measure of the diffusion perpendicular to the axon, where higher RD may represent myelin loss, axonal loss, reduced axonal packing density, or some combination of the three [33].

Quantitative anisotropy (QA) is a newer diffusion MRI metric derived from generalized-q-sampling imaging which describes the Fourier transform between water’s diffusion and signal decay [38]. QA utilizes the spin distribution function to calculate anisotropy and may offer improvements over traditional FA approaches in describing axonal loss [38]. QA has shown improvements in dealing with the noise associated with DWI scans and may offer improvements in dealing with crossing fibers and complex fiber orientations, a known issue associated with FA [39].

Correlational fiber tractography is a group level analysis technique where a DTI metric is evaluated in relation to a study variable [40]. Correlational tractography approaches have shown the potential to be more sensitive than conventional tractography approaches [41, 42]. To the best of our knowledge, this is the first study investigating correlational fiber tractography in a lysosomal storage disorder. We assessed differences between GM2 gangliosidosis disease subtypes in brain neuronal tracts of late-onset patients. DTI and correlational tractography findings from this study could be applied as outcome imaging markers for assessing interventions such as substrate reduction therapy, enzyme replacement, or gene therapy.

## Methods

### The natural history of GM2 gangliosidosis

Participants from the National Human Genome Research Institute (NHGRI) study, the “Natural History of Glycosphingolipid & Glycoprotein Storage Disorders” with a diagnosis of either LOTS or LOSD disease and at least one T1-weighted or DWI scan were included in this analysis (NCT00029965) [43]. The NIH Institutional Review Board approved this protocol (02-HG-0107). Informed consent was completed with all patients prior to participation and all research was completed in accordance with the Declaration of Helsinki. All patient visits were conducted at the National Institutes of Health Clinical Center (Bethesda MD) between 2010 and 2020.

**Fig. 1:**
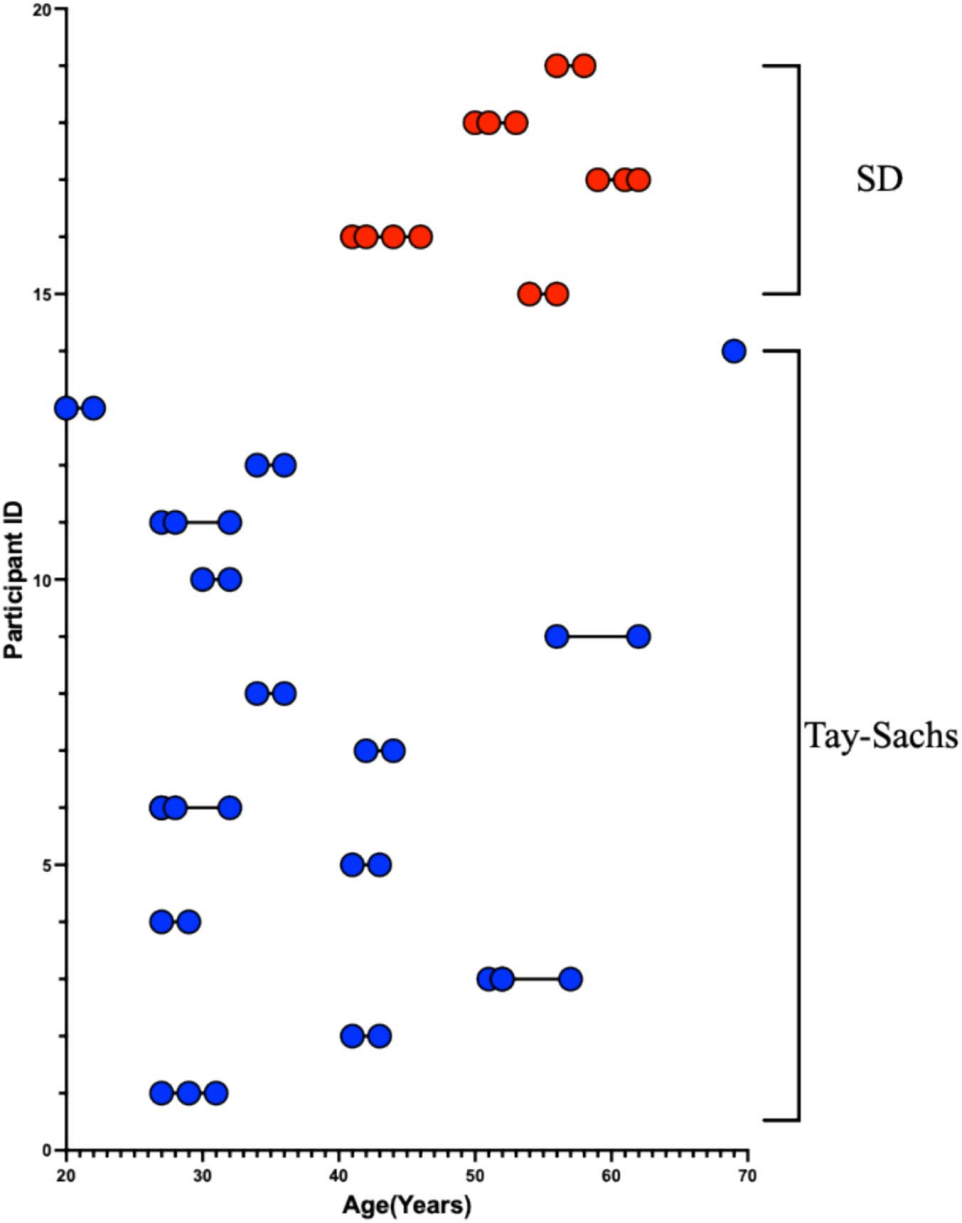
Participant age at each T1-weighted MRI Scan. LOTS patients are shown in blue and LOSD Patients are shown in red. Each T1-weighted scan is represented as a circle for all 51 scans where each of the 19 participants is on a separate row

The distinction between LOTS and LOSD was made based on differences between β-hexosaminidase A and total β-hexosaminidase (β-hexosaminidase A + β-hexosaminidase B) activity patterns and biallelic variants in either *HEXA* or *HEXB*. Fourteen LOTS and five LOSD participants were included (Fig. 1, Supplement A).

### T1-weighted MRI acquisition and analysis

51 T1-weighted magnetic resonance imaging (MRI) scans from 19 GM2 Natural History participants were acquired on a Phillips Achieva 3 T system with an 8-channel SENSE head coil. Sagittal MRI images were acquired with a 3D magnetization-prepared rapid acquisition with gradient echo (MPRAGE) sequence with the following parameters: TR/TE = 8/4 ms, slice thickness = 1 mm, flip angle = 8 degrees, NEX = 1, FOV = 220 mm. Volumetric analysis of MRI data was performed using Freesurfer’s (v7.4.1) *recon-all* reconstruction pipeline to calculate volumes of the gray matter, white matter, cerebellum (including bilateral gray and white matter), lateral ventricles, 4 th ventricle, brainstem, caudate, total intracranial volume (ICV), and thalamus [44–52].

### Neurotypical controls

1033 neurotypical control T1-weighted MRI scans were acquired from the open-source data repository, OpenNeuro. Volumetric analysis of neurotypical control data was also performed using Freesurfer’s (v7.4.1) *recon-all* reconstruction pipeline to calculate volumes for the same structures as GM2 gangliosidosis participants [44–52]. (For more information on neurotypical control datasets, see section B of the Supplementary Materials).

### DWI acquisition and preprocessing

40 DWI scans were acquired from 16 GM2 patients on a Phillips Achieva 3 T system with an 8-channel SENSE head coil. DWI were acquired with the following parameters: TR/TE = 6400/100 ms, 32-gradient encoding directions, *b*-values = 0 and 1000 s/mm^2^, voxel size = 1.875 mm × 1.875 mm × 2.5 mm, slice thickness = 2.5 mm, acquisition matrix = 128 × 128, NEX = 1, FOV = 24 cm. DWI were preprocessed using MRtrix3’s (MRtrix, v3.0.4) [53] *dwifslpreproc* [54–56] command utilizing the *dwi2 mask* [57] function followed by FSL’s (FSL, v6.0.5) *eddy* [55] and *topup* [55, 56] functions. Preprocessed data were imported into DSI Studio (DSI Studio, v2023), where imaging was quality checked for bad slices, a U-Net mask was created, and generalized q-sampling imaging (GQI) based reconstruction was performed with a diffusion sampling length ratio of 1.25 [58].

### DTI analysis

The 40 preprocessed and reconstructed DWI scans were analyzed with a diffusion MRI connectometry in DSI Studio to map fiber tracts and calculate diffusion tensor metrics along identical pathways. An atlas based deterministic fiber tractography was performed for the whole brain, cerebellum (bilateral), inferior cerebellar peduncle (bilateral), middle cerebellar peduncle (MCP), superior cerebellar peduncle (SCP), vermis, corpus callosum, and arcuate fasciculus (bilateral) and FA and MD were calculated. The angular threshold was 0 (random), the step size was 0 (random) mm. Tracks < 20 mm or > 200 mm were discarded, and 1,000,000 seeds were placed.

### Correlational tractography analysis

A separate diffusion MRI connectometry analysis was also performed in DSI Studio (DSI Studio, v2023) to identify differences in FA and MD between LOSD (n = 4) and LOTS (n = 12) patients. Correlational tractography was performed on the 16 preprocessed and reconstructed baseline scans of GM2 patients with a *T*-score between 2.0 and 4.0 for deterministic fiber tractography. A fiber tract length threshold between 10 and 40 voxels was also applied where fiber tracts shorter than the threshold were removed. The effects of age removed using a multiple regression model, and the false detection rate (FDR) was estimated using 4,000 random permutations and a threshold of < 0.05 was applied to only include axonal loss.

### Statistical analysis

Statistical analysis in this study was performed in R (The R Foundation, v4.3.1). Between group analysis was performed using linear mixed effects models with age (a fixed effect) as a covariate of no interest to evaluate T1-weighted and DTI data. For volumetric comparisons between late onset GM2 patients and neurotypical controls, late-onset GM2 patients were assigned a value of 1 and NC were assigned a value of 0 to test the effects of GM2 on the volumetric results. For comparisons between LOTS patients and NC, LOTS were assigned a value of 1 and NC were assigned a value of 0 to test the effects of LOTS on the volumetric results. For comparisons between LOSD patients and NC, LOSD were assigned a value of 1 and NC were assigned a value of 0 to test the effects of LOSD on the volumetric results. For comparisons between LOSD and LOTS, LOTS patients were assigned a value of 1 and LOSD patients were assigned a value of 0 to test distinctions in the two diseases. A subject level random intercept was used to account for repeated T1 scans conducted for each GM2 participant. *p *values < 0.05 were designated as significant for volumetric evaluations after a Bonferri correction for multiple comparisons. DTI metrics including FA, MD, RD, AD, and QA were evaluated between GM2 gangliosidosis disease subtypes to determine if there was a significant difference between LOTS and LOSD patients. A subject level random intercept was used to account for repeated DWI scans conducted for each participant. *p* values < 0.05 were considered significant.

## Results

### Volumetric MRI analysis

**Fig. 2:**
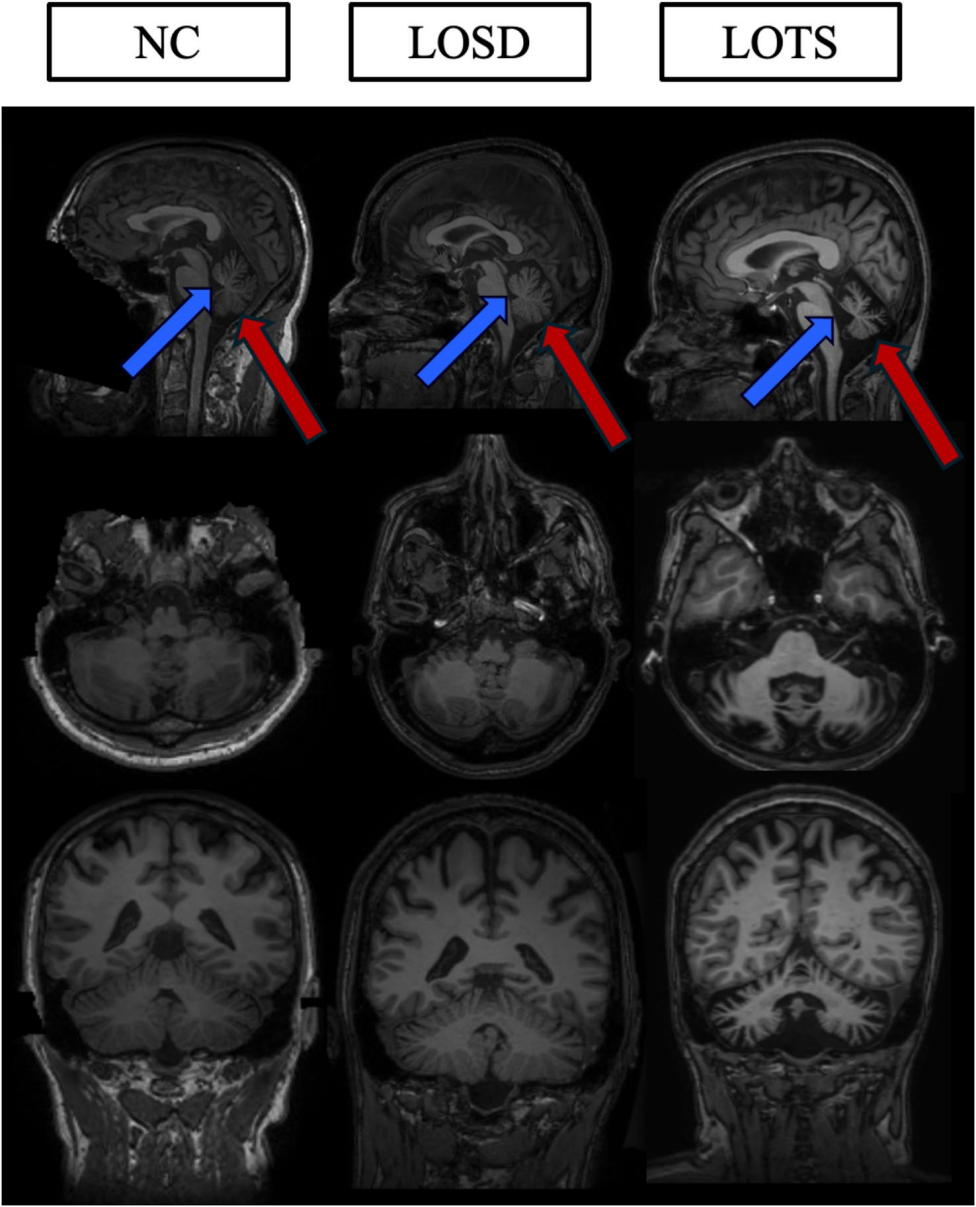
T1-weighted MRI comparisons. One age matched neurotypical control (NC, 55-year-old male), one age matched LOSD patient (56-year-old male), and one age matched LOTS patient (56-year-old female) shown in the Sagittal, Axial, and Coronal planes. The blue arrows designate the 4 th ventricle for each participant and the red arrows designate the cerebellum for each participant. The LOTS patient shows significant cerebellar atrophy in all three views. The neurotypical control was from the NIMH dataset

**Fig. 3:**
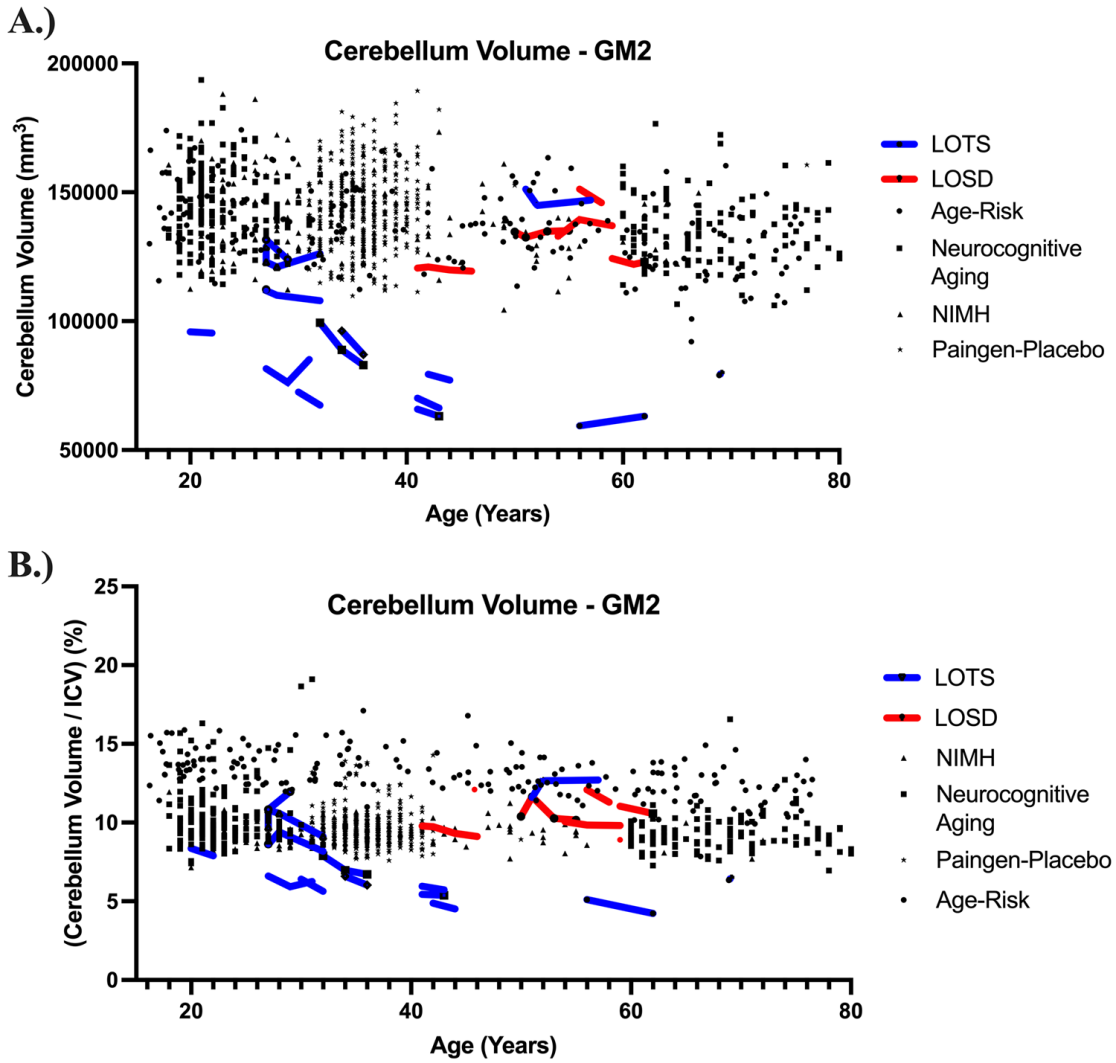
Age related changes in cerebellum volume. LOSD (red) participants demonstrate normal cerebellum volume when compared with neurotypical age matched controls. LOTS participants (blue) demonstrate significantly diminished cerebellum volume when compared with neurotypical controls. **A** Total cerebellum volume including bilateral cerebellum gray and white matter. **B** Cerebellum volumes were normalized to total intracranial volume and are expressed as a percentage. Statistical differences were observed between GM2 patients and neurotypical controls (*p*_corrected_ < 0.01), LOTS patients and neurotypical controls (*p*_corrected_ < 0.01), and between LOTS patients and LOSD patients (*p*_corrected_ < 0.01) for cerebellar volume

Figure 2 demonstrates the cerebellar atrophy and associated 4 th ventricle enlargement associated with LOTS. Furthermore, Table 1 describes the volumetric findings between neurotypical controls (NC, *n* = 1033), GM2 gangliosidosis patients (*n* = 19), LOSD (*n* = 5), and LOTS (*n* = 14) participants as evaluated by linear mixed effects modeling. Late onset GM2 patients had significantly smaller cerebellum (*p*_corrected_ < 0.01) and enlarged 4 th ventricle volume compared to NC (*p*_corrected_ < 0.01) when controlled for ICV (Fig. 3). Late onset GM2 patients had significantly smaller cerebellar gray matter than NC, however cerebellar white matter was not statistically different. Furthermore, LOTS patients had significantly smaller cerebellum volume both bilaterally and affecting both gray and white matter when compared with NC. LOTS patients also had enlargement of the 4 th ventricle (*p*_corrected_ < 0.01) compared with NC. No other measurements showed a difference between the two subtypes. There was no statistical difference in any of our volumetric analysis between LOSD patients and NC.

**Table 1:** T1-weighted volumetric MRI analysis. All volumes were controlled for ICV, apart from ICV. Statistical analysis was performed using a linear mixed effects model (LMEM) where *p*-values < 0.05 after a Bonferri correction for multiple comparisons were considered significant and bolded. Estimates and standard errors from the can be found in Supplementary Table E1

Structure	*GM2 v NC*	*LOSD v NC*	*LOTS v NC*	*LOSD v LOTS*
*χ*^2^(1) *p *value *p *value (corrected)				
Gray matter volume	0.14 0.71 1.00	0.14 0.71 1.00	0.05 0.83 1.00	2.33 0.13 0.51
White matter volume	0.35 0.56 1.00	1.13 0.29 1.00	< 0.01 0.97 1.00	4.87 0.03 0.11
Cerebellum	22.41 ** < 0.01** ** < 0.01**	0.23 0.63 1.00	34.05 ** < 0.01** ** < 0.01**	**11.08** ** < 0.01** ** < 0.01**
Left cerebellar white matter	4.69 0.03 0.12	2.21 0.14 0.55	11.82 ** < 0.01** ** < 0.01**	13.60 ** < 0.01** ** < 0.01**
Right cerebellar white matter	4.67 0.03 0.12	1.95 0.16 0.65	11.39 ** < 0.01** ** < 0.01**	10.24 ** < 0.01** ** < 0.01**
Left cerebellar gray matter	24.74 ** < 0.01** ** < 0.01**	0.12 0.73 1.00	36.43 ** < 0.01** ** < 0.01**	9.49 ** < 0.01** ** < 0.01**
Right cerebellar gray matter	29.19 ** < 0.01** ** < 0.01**	0.01 0.94 1.00	39.65 ** < 0.01** ** < 0.01**	10.23 ** < 0.01** ** < 0.01**
Ventricles	0.56 0.45 1.00	0.02 0.90 1.00	0.65 0.42 1.00	2.26 0.13 0.53
4th ventricle	20.27 ** < 0.01** ** < 0.01**	1.623 0.20 0.81	37.78 ** < 0.01** ** < 0.01**	6.633 **0.01** **0.04**
Thalamus	2.97 0.08 0.34	0.03 0.87 1.00	3.72 0.05 0.20	6.50 **0.01** **0.04**
Caudate	3.83 0.05 0.20	0.43 0.51 1.00	3.62 0.06 0.23	0.20 0.66 1.00
Intracranial volume (ICV)	6.04 0.01 0.06	1.29 0.26 1.00	4.76 0.03 0.12	0.51 0.48 1.00
Brainstem	1.13 0.29 1.00	0.10 0.75 1.00	1.12 0.29 1.00	2.69 0.10 0.40

LOTS patients also had significantly smaller cerebellum volume both bilaterally and affecting both gray and white matter when compared with LOSD patients. 4 th ventricle volume was evaluated to be different significantly higher in LOTS patients compared to LOSD patients (*p*_corrected_ = 0.04). Thalamic volume was evaluated to be smaller in LOTS patients compared to LOSD patients (*p*_corrected_ = 0.04). No other measurements showed a difference between the two subtypes.

### Diffusion tensor imaging analysis—fractional anisotropy (FA)

**Fig. 4:**
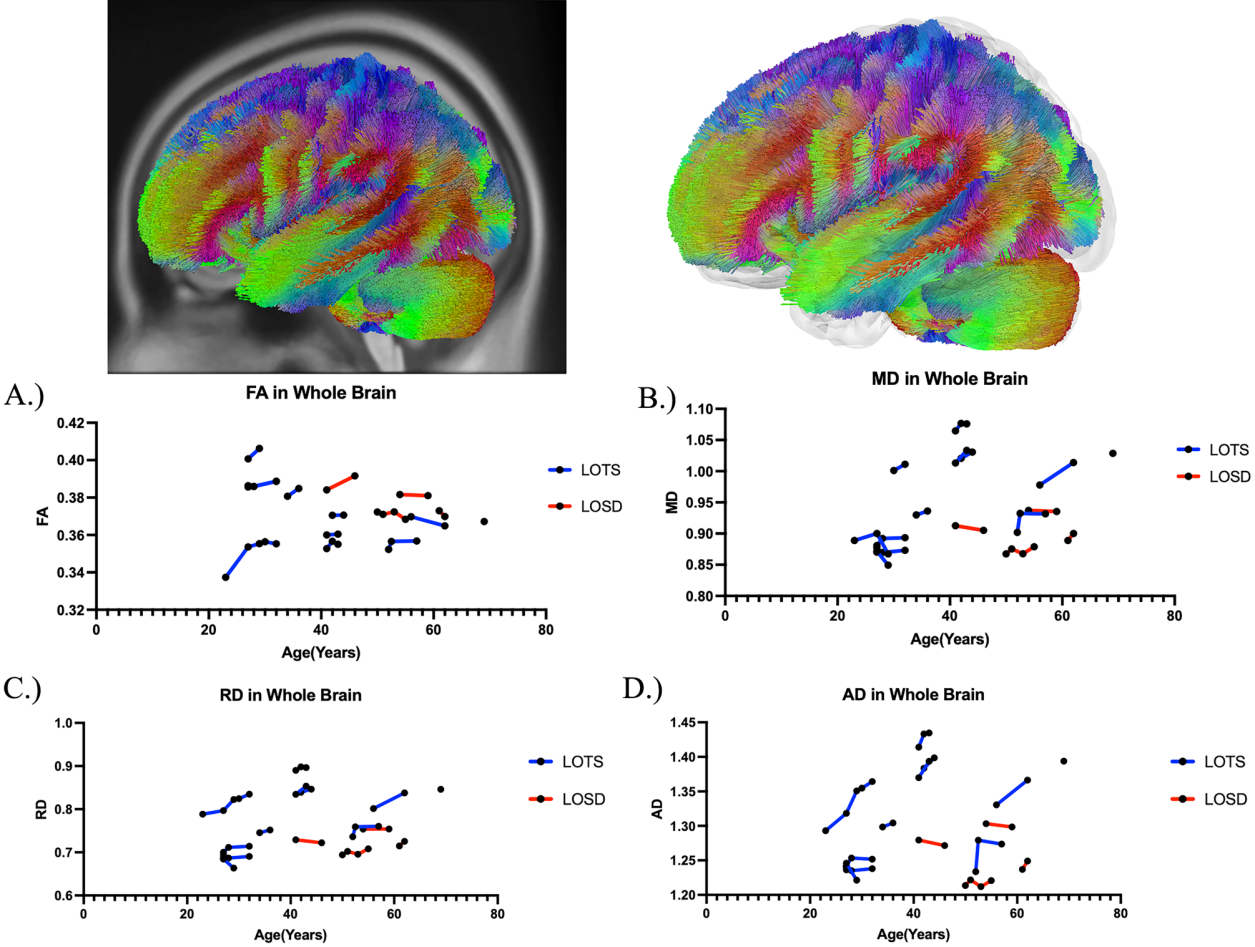
Atlas Based Fiber Tractography of the whole brain demonstrating age related effects on **A** fractional anisotropy (FA), **B** mean diffusivity (MD), **C** radial diffusivity (RD), **D** axial diffusivity (AD) between LOTS patients (blue) and LOSD patients (red). LOTS patients demonstrated no difference in FA (*χ*^2^(1) = 0.70, *p* = 0.40), and increased MD (*χ*^2^(1) = 7.62, *p* < 0.01), RD (*χ*^2^(1) = 7.28, *p* < 0.01), and AD (*χ*^2^(1) = 7.86, *p* < 0.01) compared to LOSD patients in fiber tracts throughout the whole brain when age was accounted for

**Fig. 5:**
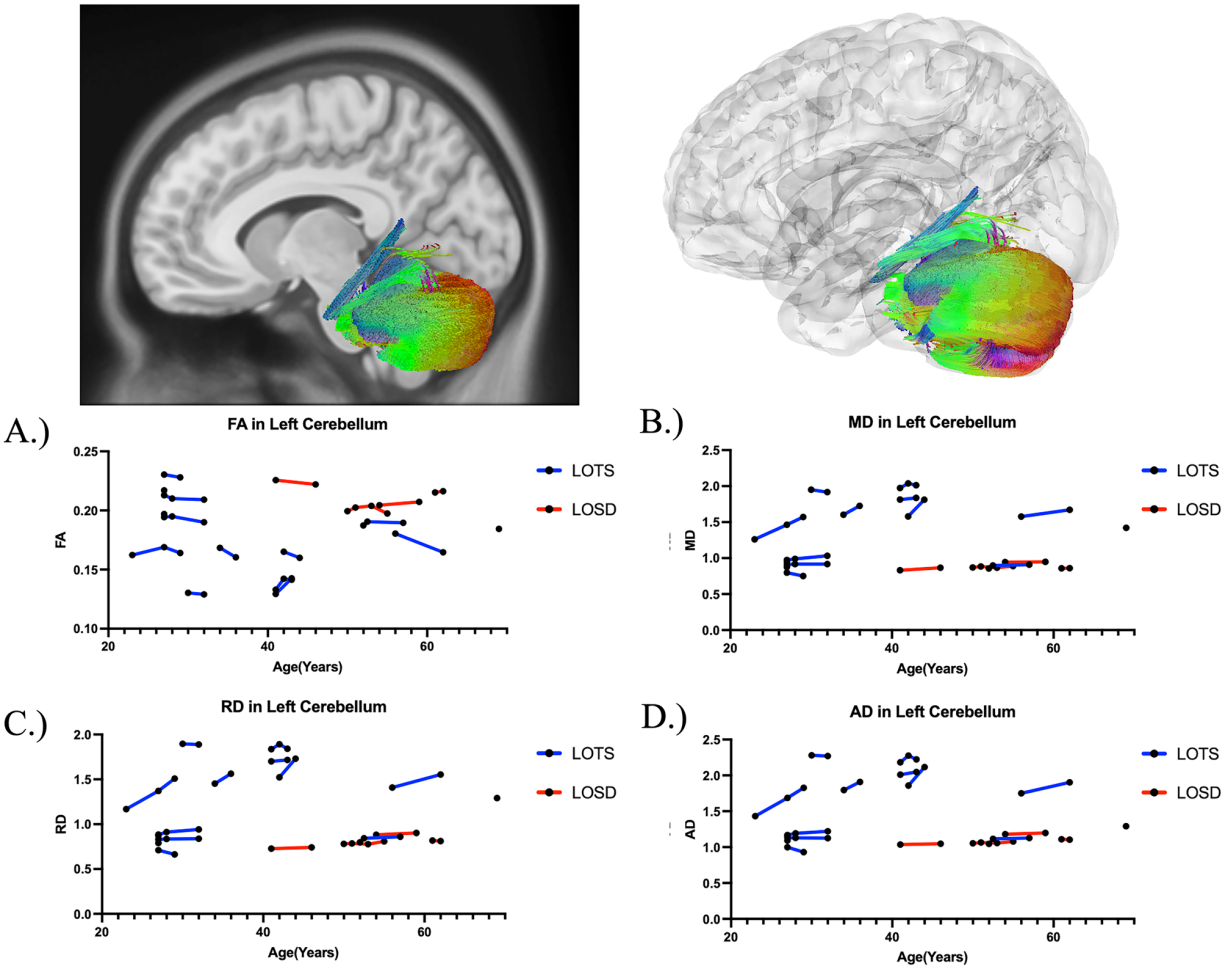
Atlas Based Fiber Tractography of the left cerebellum demonstrating age related effects on **A** fractional anisotropy (FA), **B** mean diffusivity (MD), **C** radial diffusivity (RD), **D** axial diffusivity (AD) between LOTS patients (blue) and LOSD patients (red). LOTS patients demonstrated decreased FA (*χ*^2^(1) = 7.70, *p* < 0.01), and increased MD (*χ*^2^(1) = 8.42, *p* < 0.01), RD (*χ*^2^(1) = 8.51, *p* < 0.01), and AD (*χ*^2^(1) = 8.25, *p* < 0.01) compared to LOSD patients in left cerebellar fiber tracts when age was accounted for

**Fig. 6:**
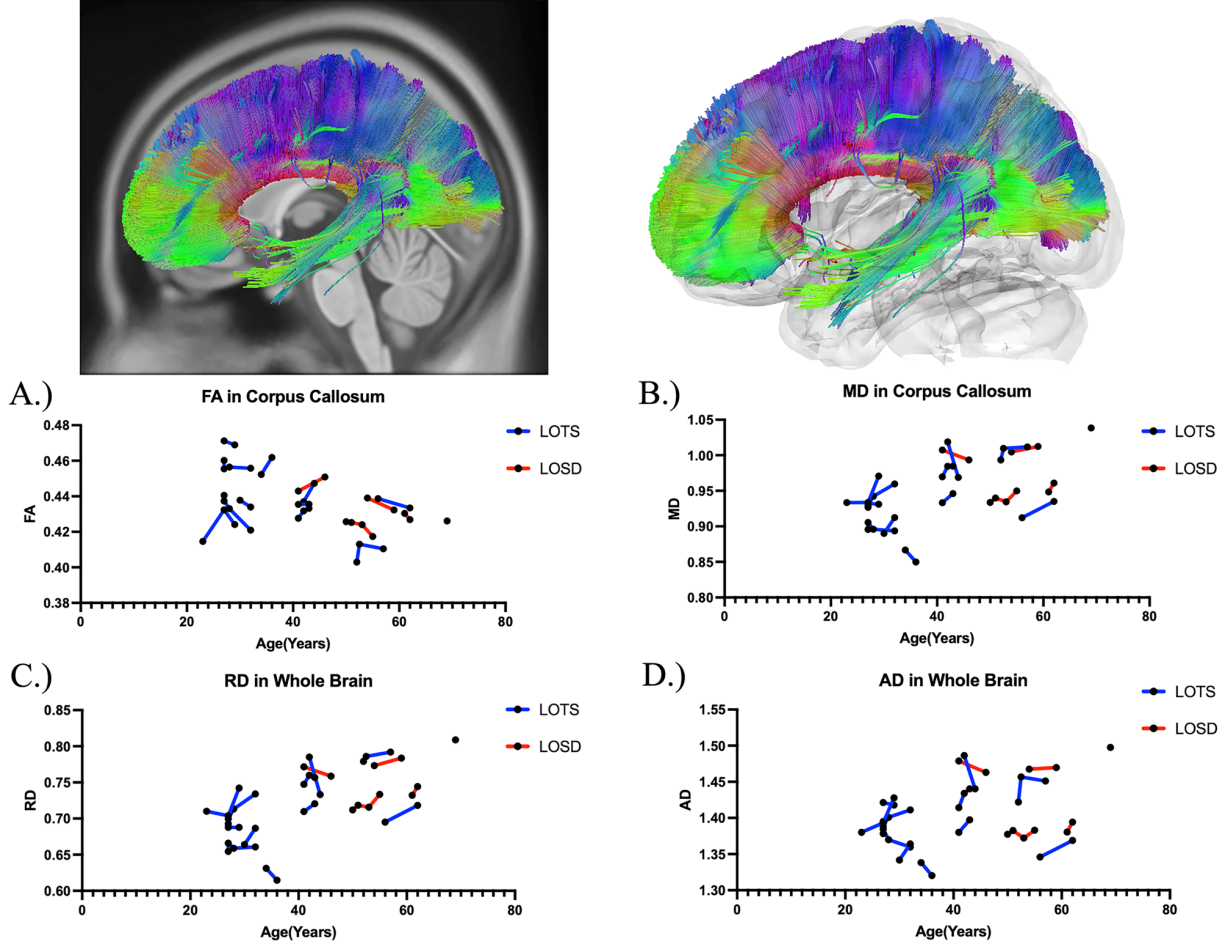
Atlas Based Fiber Tractography of the corpus callosum demonstrating age related effects on **A** fractional anisotropy (FA), **B** mean diffusivity (MD), **C** radial diffusivity (RD), **D** axial diffusivity (AD) between LOTS patients (blue) and LOSD patients (red). There were no significant differences in between LOTS and LOSD for FA, MD, RD, or AD in the corpus callosum when age was accounted for

Table 2 summarizes the DTI results of FA between LOTS and LOSD patients as evaluated by linear mixed effects modeling. There was no statistical difference in FA between LOTS and LOSD patients throughout the whole brain (Fig. 4A), corpus callosum (Fig. 6A), or bilateral arcuate fasciculus (Supplement Figure D7A and D8A). There was also no statistical difference in FA in the bilateral inferior cerebellar peduncle and middle cerebellar peduncle in LOSD patients compared to LOTS patients. LOSD patients had higher FA in the bilateral cerebellum (Fig. 5A, Supplement Figure D1A), superior cerebellar peduncle (Supplement Figure D5A), and vermis (Supplement Figure D6A) compared to LOTS patients.

**Table 2:** Diffusion tensor imaging results of FA in Atlas Fiber tractography pathways evaluating differences between LOTS and LOSD patients. Estimates and standard errors were calculated from the linear mixed effects model evaluating the differences between LOTS and LOSD. *P*-values less than 0.05 were considered significant and bolded

Pathway	Estimate	Standard error	*χ*^2^(1)	*p* value (>* χ*^2^)
Whole brain	0.0070	0.0094	0.70	0.40
Left cerebellum	0.0479	0.0167	7.70	** < 0.01**
Right cerebellum	0.0391	0.0166	5.50	**0.02**
Left inferior cerebellar peduncle	0.0380	0.0222	3.10	0.08
Right inferior cerebellar peduncle	0.0250	0.0190	1.89	0.17
Middle cerebellar peduncle	0.0223	0.0183	1.65	0.20
Superior cerebellar peduncle	0.0268	0.0121	4.80	**0.03**
Vermis	0.0498	0.0223	5.05	**0.02**
Corpus callosum	0.0016	0.0087	0.05	0.82
Left arcuate fasciculus	0.0090	0.0104	0.92	0.34
Right arcuate fasciculus	−0.0007	0.0124	0.04	0.83

### Diffusion tensor imaging analysis—mean diffusivity (MD)

Table 3 summarizes the DTI results of MD between LOTS and LOSD patients as evaluated by linear mixed effects modeling. MD was higher in white matter pathways throughout the whole brain (Fig. 4B), bilateral cerebellum (Fig. 5B and Supplement Figure D1B), bilateral inferior cerebellar peduncle (Supplement Figure D2B and D3B), MCP (Supplement Figure D4B), SCP (Supplement Figure D5B), and vermis (Supplement Figure D6B) in LOTS patients compared to LOSD patients. MD in the corpus callosum (Fig. 6B) and bilateral arcuate fasciculus (Supplement Figures D7B and D8B) were not different between LOTS and LOSD patients.

**Table 3:** Diffusion tensor imaging results of MD in Atlas Fiber tractography pathways evaluating differences between LOTS and LOSD patients. Estimates and standard errors were calculated from the linear mixed effects model evaluating the differences between LOTS and LOSD. *P*-values less than 0.05 were considered significant and bolded

Pathway	Estimate	Standard error	*χ*^2^(1)	*p* value (>* χ*^2^)
Whole brain	−0.0967	0.0337	7.62	** < 0.01**
Left cerebellum	−0.7262	0.2385	8.42	** < 0.01**
Right cerebellum	−0.7157	0.2316	8.56	** < 0.01**
Left inferior cerebellar peduncle	−0.5015	0.1837	7.02	** < 0.01**
Right inferior cerebellar peduncle	−0.4447	0.1441	8.55	** < 0.01**
Middle cerebellar peduncle	−0.3459	0.1302	6.81	** < 0.01**
Superior cerebellar peduncle	−0.2665	0.0962	7.19	** < 0.01**
Vermis	−0.7146	0.2786	6.62	**0.01**
Corpus callosum	0.0028	0.0248	0.02	0.90
Left arcuate fasciculus	−0.0231	0.0150	2.65	0.10
Right arcuate fasciculus	−0.0027	0.0135	0.12	0.73

**Fig. 7:**
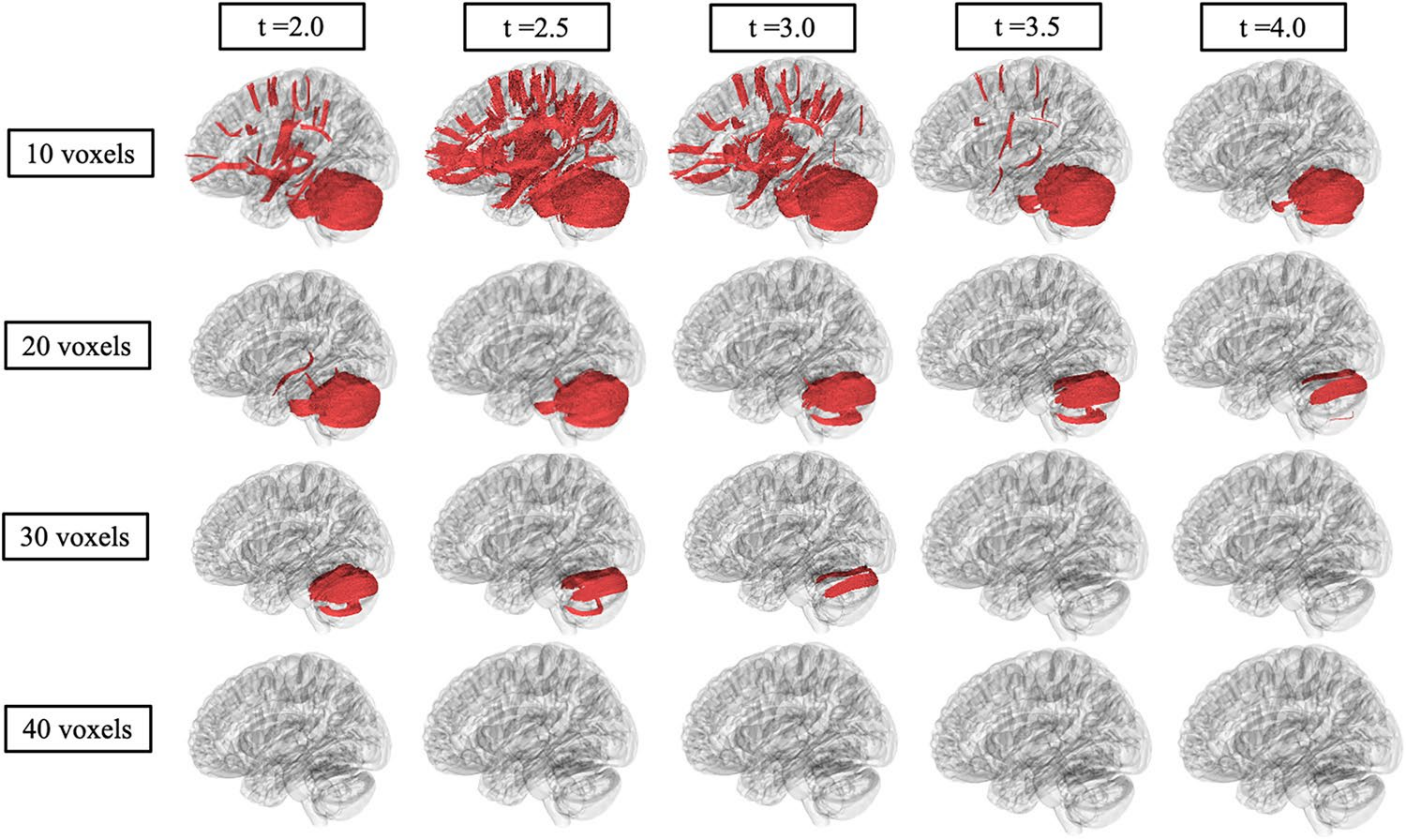
Correlational fiber tractography assessed differences in fractional anisotropy in LOSD and LOTS patients at varying length (voxels) and T thresholds. Fiber tracts shown in red were evaluated to have a higher fractional anisotropy in LOSD patients compared to LOTS patients and were observed primarily in the cerebellum (FDR < 0.05). No fiber tracts were evaluated to have a higher FA in LOTS patients compared to LOSD patients (blue)

**Fig. 8:**
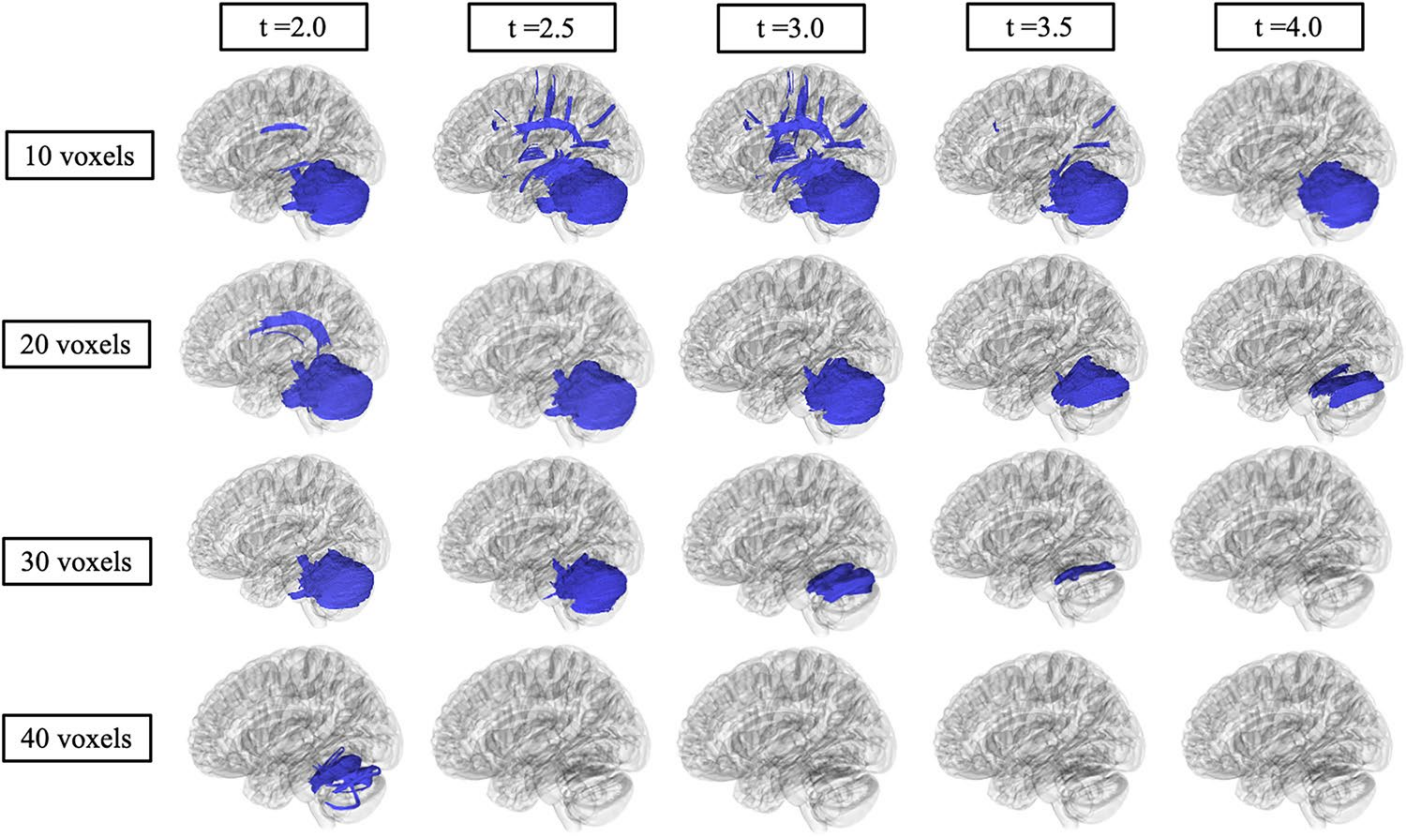
Correlational fiber tractography assessed differences in mean diffusivity in LOSD and LOTS patients at varying length (voxels) and T thresholds. Fiber tracts shown in blue were evaluated to have a higher mean diffusivity in LOTS patients compared to LOSD patients and were observed primarily in the cerebellum (FDR < 0.05). No fiber tracts were evaluated to have a higher mean diffusivity in LOSD patients compared to LOTS patients (red)

### Diffusion tensor imaging analysis—radial diffusivity (RD)

Supplement Table F1 summarizes the DTI results of RD between LOTS and LOSD patients as evaluated by linear mixed effects modeling. RD was higher in white matter pathways throughout the whole brain (Fig. 4C), bilateral cerebellum (Fig. 5C and Supplement Figure D1C), bilateral inferior cerebellar peduncle (Supplement Figure D2C and D3C), MCP (Supplement Figure D4C), SCP (Supplement Figure D5C), and vermis (Supplement Figure D6C) in LOTS patients compared to LOSD patients. RD in the corpus callosum (Fig. 6C) and bilateral arcuate fasciculus (Supplement Figure D7C and D8C) were not different between LOTS and LOSD patients.

### Diffusion tensor imaging analysis—axial diffusivity (AD)

Supplement Table F2 summarizes the DTI results of AD between LOTS and LOSD patients as evaluated by linear mixed effects modeling. AD was higher in white matter pathways throughout the whole brain (Fig. 4D), bilateral cerebellum (Fig. 5D and Supplement Figure D1D), bilateral inferior cerebellar peduncle (Supplement Figure D2D and D3D), MCP (Supplement Figure D4D), SCP (Supplement Figure D5D), and vermis (Supplement Figure D6D) in LOTS patients compared to LOSD patients. AD in the corpus callosum (Fig. 6D) and bilateral arcuate fasciculus (Supplement Figure D7D and D8D) were not different between LOTS and LOSD patients.

### Diffusion tensor imaging analysis—Quantitative anisotropy (QA)

Supplement Table F3 summarizes the DTI results of QA between LOTS and LOSD patients as evaluated by linear mixed effects modeling. QA was not statistically different between LOTS and LOSD patients for fiber tracts throughout the whole brain, bilateral arcuate fasciculus, bilateral cerebellum, corpus callosum, vermis, MCP, SCP, or bilateral inferior cerebellar peduncle.

### Correlational fiber tractography analysis

Figures 7 and 8 show significant differences in FA and MD in neuronal fiber tracts between LOSD and LOTS patients as evaluated by correlational fiber tractography, respectively. When correlated to disease subtype, fiber tracts with higher FA and lower MD were identified exclusively in patients with LOSD, most notably in cerebellar pathways. No fiber tracts were evaluated to have higher FA or lower MD in LOTS patients when compared to LOSD patients. Higher T-score and length thresholds demonstrated less results and ultimately no results at the highest thresholds for FA and MD.

Correlational fiber tractography results for AD, RD, and QA are shown in section G of the Supplement Materials. AD and RD results were similar to those observed with MD where LOTS patients were observed to have higher AD and RD in fiber tracts in the cerebellum. QA results were similar to those observed with FA where fiber tracts with higher QA in LOSD patients were identified in the cerebellum. QA also highlighted fiber tracts in the brainstem with higher QA in LOSD patients, along with a few sparse fiber tracts with higher QA in LOTS located around the corpus callosum. Higher T-score and length thresholds demonstrated less results and ultimately no results at the highest thresholds for QA, AD and RD.

## Discussion

In this study, we aimed to discern differences in adult-onset GM2 gangliosidosis disease subtypes through T1-weighted volumetric and DWI-derived analysis. Our T1-weighted volumetric analysis first demonstrated the distinctions in cerebellar volume (Fig. 3) and 4th ventricle volume between late-onset GM2 gangliosidosis patients and neurotypical controls. Furthermore, we demonstrated how LOTS patients, when considered alone also have a worsening cerebellar and 4th ventricle pathology than neurotypical controls. There was no statistical difference between LOSD and neurotypical controls for any volumetric measurements.

LOTS patients were also found to have decreased cerebellar volume (including both gray and white matter), thalamic volume, and cerebellar atrophy-associated enlargement of the 4th ventricle compared to LOSD. Our volumetric work validates and expands on the findings previously described in Rowe et al. [24], however, their study did not expand into DTI.

Cerebellar atrophy in late-onset GM2 gangliosidosis patients has been previously correlated to both the Friedreich Ataxia Rating Scale (FARS) and the brief ataxia rating scale (BARS) demonstrating that patients with more severe ataxic symptoms had corresponding severe atrophy of the cerebellum [24]. Furthermore, one study which segmented the cerebellum into distinct lobules found that ataxia symptoms were related to more significant cerebellar atrophy across many cerebellar lobules in LOTS patients compared to controls [59]. However, in this study, tremors, psychiatric symptoms, and upper motor neuron signs were not found to correlate with cerebellar lobule atrophy suggesting the underlying disease pathology requires further explanation. The thalamic volume differences between LOTS and LOSD provide an interesting result. While atrophy of the ventral and lateral thalamic motor nuclei has been previously shown in LOTS patients compared to controls [59], we observed no statistical difference between LOTS patients and controls for total thalamic volume. Future investigations into this relationship using analysis techniques capable of segmenting the nuclei thalamus are warranted.

Through our DTI analysis, we further supported the finding of more severe cerebellar involvement in LOTS patients when compared to LOSD patients. In 4 out of 7 cerebellar white matter pathways, we found LOTS patients had reduced FA compared to LOSD patients (Table 1). In all 7 cerebellar pathways, LOTS patients also had increased MD, RD, and AD compared to LOSD patients (Table 3, Supplement Tables F1 and F2). The model free metric QA did not show any significant differences in the 7 cerebellar pathways, the whole brain, or the corpus callosum between LOTS and LOSD patients (Supplement Table F3).

Our correlational fiber tractography results further demonstrate the cerebellar differences between LOTS and LOSD patients. LOTS patients were observed to have higher MD (Fig. 8) in cerebellar fiber tracts compared to LOSD. AD and RD (Supplement Figures G1 and G2) correlational fiber tractography results were similar to MD results as both AD and RD were higher in LOTS patients compared to LOSD primarily in cerebellar pathways. LOSD patients were observed to have higher FA (Fig. 7) in cerebellum tracts compared to LOTS patients. QA results (Supplement Figure G3) were similar to our FA result where QA was observed to be higher in LOSD patients compared to LOTS patients in cerebellar neuronal pathways. Our QA correlational fiber tractography results also highlight brainstem involvement with lower QA in LOTS patients when compared with LOSD patients which was not observed with any of our DTI metrics (FA/MD/AD/RD).

To determine the sensitivity of our correlational fiber tractography results we tested T-scores from 2.0 to 4.0 and length thresholds from 10 to 40 voxels for all five of our diffusion MRI metrics (QA/FA/MD/AD/RD). As expected, we found significantly fewer correlational tractography results when these thresholds were increased. Higher T-scores are evaluated to have stronger correlations with the study variable, in our case evaluating GM2 gangliosidosis disease subtype. We found significant results in the cerebellum with T-scores between 2.0 and 4.0 for all five of our diffusion MRI metrics as evaluated by correlational tractography when the length threshold was low (10 voxels). When the length threshold was increased, correlational fiber tractography found notably fewer results and ultimately almost no results at the highest length threshold (40 voxels). Previous studies utilizing correlational fiber tractography have utilized T-scores as low as 2.0 and length thresholds as low as 20 voxels to demonstrate correlations with their study variable [60]. However, correlational fiber tractography T-score and length thresholds in relation to differentiating disease subtypes requires further investigation. Our results also suggest correlational tractography may be an important tool in distinguishing between diseases and disease subtypes with similar neurologic clinical presentations or phenotypes.

Limitations of this study need to be considered before these methods and results are used to guide clinical practice or a clinical trial. First, this study is limited by a small sample size (n = 19), with five LOSD patients and 14 LOTS patients with long disease course. Future studies investigating distinctions between LOTS and LOSD patients should include a larger cohort and the addition of neurotypical controls to determine if LOSD patients might also have subtle cerebellar pathologic findings. Similarly, comparison of tractography patterns between LOTS and other forms of monogenic cerebellar degeneration might be informative and relevant to phenotypic features unique to LOTS disease. Second, the use of FA, and particularly both AD and RD as biomarkers for white matter disease have been met with skepticism [61]. Voxels with crossing white matter fibers and complex fiber geometry can lead to incorrect interpretations of these metrics when taken in isolation [61]. The present study addresses this issue by first taking into consideration MD which is more robust than FA, AD, and RD. Second, these metrics were not interpreted in isolation, and they supported the same conclusion of distinct cerebellar pathology in LOTS patients when compared to LOSD patients. Lastly, the present study focuses on T1-weighted volumetric analysis and DTI, where future studies should investigate neurite orientation dispersion and density imaging (NODDI) [62], metabolic activity diffusion imaging (MADI) [63], and magnetic resonance spectroscopy (MRS) to define the full neuroimaging phenotypic range of late-onset GM2 gangliosidosis patients.

## Conclusion

In this study, we aimed to describe differences in volumetric and diffusion MRI metrics between LOSD and LOTS patients. Our DTI analysis found novel differences between LOTS and LOSD patients primarily in cerebellar pathways in FA, MD, RD, and AD, suggesting altered cerebellar white matter structural integrity in LOTS patients. To our knowledge, this is the first study using correlational tractography in a lysosomal storage disorder to demonstrates differences in disease subtypes. FA decreases and MD, AD, and RD increases were observed in LOTS patients when compared to LOSD patients in cerebellum fiber tracts which further supports the result of distinct or at least more severe cerebellar pathology in LOTS patients. This is consistent with cerebellar deficits being more common in LOTS patients. The exact molecular mechanism by which some phenotypic dichotomy between LOTS and LOSD exists remains a matter of great interest. These findings also suggest that in the context of clinical trials, outcome parameters should consider factoring these differences.

## Supplementary Information

Below is the link to the electronic supplementary material.

Supplementary file1 (DOCX 13832 KB)

## Data Availability

The data described in this manuscript are available from the corresponding author upon reasonable request.

## Abbreviations

AD: Axial diffusivity
DTI: Diffusion tensor imaging
DWI: Diffusion weighted imaging
FA: Fractional anisotropy
ICV: Intracranial volume
LOSD: Late-onset Sandhoff disease
LOTS: Late-onset Tay–Sachs
MCP: Middle cerebellar peduncle
MD: Mean diffusivity
MRI: Magnetic resonance imaging
MRS: Magnetic resonance spectroscopy
NC: Neurotypical control
QA: Quantitative anisotropy
RD: Radial diffusivity
SCP: Superior cerebellar peduncle
