# Supplementary material for: Late-onset GM2 gangliosidosis: magnetic resonance imaging, diffusion tensor imaging, and correlational fiber tractography differentiate Tay–Sachs and Sandhoff diseases
Source: J Neurol. 2025 Apr 23;272(5):355. doi: 10.1007/s00415-025-13091-3 (PMC12018622; doi:10.1007/s00415-025-13091-3)
Supplement: Supplementary file 1 — Supplementary file1 (DOCX 13832 KB) [file 415_2025_13091_MOESM1_ESM.docx]

**Late-onset GM2 Gangliosidosis: Magnetic resonance imaging, diffusion tensor imaging, and correlational fiber tractography differentiate Tay-Sachs and Sandhoff Diseases**

**Supplementary Material**

Table of Contents

**Methods**……………………………………………………………………………………..….…*2*

Supplement A: Natural History Study Participant Characteristics……………..………...…..……*2*

Supplement B: Neurotypical Controls Participants and T1-Weighted Acquisition…….....………*6*

**Results**……………………………………………………………………………..……………...*9*

Supplement C: T1-Weighted Volumetric Analysis………………………………………...…..….*9*

Supplement D: Diffusion Tensor Imaging Analysis……………………………………………...*21*

Supplement E. Estimates and Standard Error of Volumetric LMEM…….…………..………….*30*

Supplement F. Diffusion Tensor Imaging Analysis of RD, AD, and QA from LMEM………….*31*

Supplement G: Correlational Fiber Tractography….………………………………………….…*33*

**Supplementary References**…………………………………………………………………..…*36*

**Supplementary Methods**

**Supplement A: Natural History Study Participant Characteristics**

**Table A1. Natural History Study T1-Weighted MRI Cohort (n = 19).** N/A are designated when a participant did not have the above referenced MRI scan.

| Participant | GM2  Sub-type | Age of Symptom Onset (years old) | Age of Diagnosis (years old) | Baseline Scan Age (years old) | Scan #2 Age (years old) | Scan #3 Age (years old) | Scan #4 Age (years old) | Average T1-weighted interval (years) | Number of T1 Scans |
| --- | --- | --- | --- | --- | --- | --- | --- | --- | --- |
| NHS 32 | LOTS | 14 | 22 | 27 | 29 | 31 | N/A | 2 | 3 |
| NHS 46 | LOTS | 8 | 26 | 41 | 43 | N/A | N/A | 2 | 2 |
| NHS 47 | LOTS | 27 | 47 | 51 | 52 | 52 | 57 | 2 | 4 |
| NHS 50 | LOTS | 8 | 21 | 27 | 29 | N/A | N/A | 2 | 2 |
| NHS 103 | LOTS | 4 | 33 | 41 | 43 | N/A | N/A | 2 | 2 |
| NHS 56 | LOTS | Early childhood | 26 | 27 | 27 | 28 | 32 | 1.67 | 4 |
| NHS 70 | LOTS | 25 | 41 | 42 | 44 | N/A | N/A | 2 | 2 |
| NHS 65 | LOTS | 10 | 34 | 34 | 36 | N/A | N/A | 2 | 2 |
| NHS 57 | LOTS | 5 | 53 | 56 | 62 | N/A | N/A | 6 | 2 |
| NHS 75 | LOSD | 12 | 30 | 54 | 56 | 59 | N/A | 2.5 | 3 |
| NHS 68 | LOSD | 37 | 40 | 41 | 42 | 44 | 46 | 1.67 | 4 |
| NHS 67 | LOTS | 10 | 33 | 30 | 32 | N/A | N/A | 2 | 2 |
| NHS 55 | LOTS | 11 | 26 | 27 | 27 | 28 | 32 | 1.67 | 4 |
| NHS 43 | LOSD | 10 | 58 | 59 | 61 | 62 | N/A | 1.5 | 3 |
| NHS 45 | LOSD | 8 | 48 | 50 | 51 | 53 | 55 | 1.67 | 4 |
| NHS 37 | LOTS | 13 | 33 | 32 | 34 | 36 | N/A | 2 | 3 |
| NHS 66 | LOTS | 10 | 20 | 20 | 22 | N/A | N/A | 2 | 2 |
| NHS 104 | LOTS | Early childhood | 67 | 69 | N/A | N/A | N/A | N/A | 1 |
| NHS 74 | LOSD | 16 | 31 | 56 | 58 | N/A | N/A | 2 | 2 |

**Figure A1. Participant age at each T1-Weighted MRI Scan.** LOTS patients are shown in blue, and LOSD patients are shown in red. Each T1-weighted scan is represented as a circle for all 51 scans where each of the 19 participants is on a separate row.

**Table A2. Natural History Study DWI Cohort (n = 16).** N/A are designated when a participant did not have the above referenced MRI scan.

| Participant | GM2  Sub-type | Baseline Age (years old) | Scan #2 Age (years old) | Scan #3 Age (years old) | Scan #4 Age (years old) | Average DWI interval (years) | Number of DWI Scans |
| --- | --- | --- | --- | --- | --- | --- | --- |
| NHS 32 | LOTS | 27 | 29 | 31 | N/A | 2 | 3 |
| NHS 46 | LOTS | 41 | 42 | 43 | N/A | 1 | 3 |
| NHS 47 | LOTS | 52 | 52 | 57 | N/A | 2.5 | 3 |
| NHS 50 | LOTS | 27 | 29 | N/A | N/A | 2 | 2 |
| NHS 103 | LOTS | 41 | 43 | N/A | N/A | 2 | 2 |
| NHS 56 | LOTS | 27 | 27 | 28 | 32 | 1.67 | 4 |
| NHS 70 | LOTS | 42 | 44 | N/A | N/A | 2 | 2 |
| NHS 65 | LOTS | 34 | 36 | N/A | N/A | 2 | 2 |
| NHS 57 | LOTS | 56 | 62 | N/A | N/A | 6 | 2 |
| NHS 75 | LOSD | 54 | 59 | N/A | N/A | 5 | 2 |
| NHS 68 | LOSD | 41 | 46 | N/A | N/A | 5 | 2 |
| NHS 67 | LOTS | 30 | 32 | N/A | N/A | 2 | 2 |
| NHS 55 | LOTS | 27 | 27 | 28 | 32 | 1.67 | 4 |
| NHS 43 | LOSD | 61 | 62 | N/A | N/A | 1 | 2 |
| NHS 45 | LOSD | 50 | 51 | 53 | 55 | 1.67 | 4 |
| NHS 104 | LOTS | 69 | N/A | N/A | N/A | N/A | 1 |

**Figure A2. Participant age at each DWI Scan.** LOTS patients are shown in blue, and LOSD patients are shown in red. Each Diffusion weighted scan is represented as a circle for all 40 scans where each of the 16 participants is on a separate row.

**Supplement B: Neurotypical Control Participants and T1-Weighted Acquisition**

*NIMH^2,3^*

Participants from “The National Institute of Mental Health (NIMH) Intramural Healthy Volunteer Dataset”^2,3^ were scanned using a General Electric (GE) discovery MR750W 3T system with a 32-channel head coil. There were 62 participants with the FSPGR sequence and 92 participants with the MPRAGE sequence.^2,3^ With the following parameters:

**MPRAGE**

TR/TE = 6.95/2.92 ms

Flip angle = 8^∘^

Voxel = 1 mm isotropic voxels

**FSPGR**

TR/TE = 7.35/3.04 ms

Flip angle = 11^∘^

Slice Thickness = 1.2 mm

*Neurocognitive Aging^4,5^*

Participants from the “Neurocognitive aging data release with behavioral, structural, and multi-echo functional MRI measures” were included in this analysis.^4,5^ MRI data was acquired from one of two sites either the Cornell Magnetic Resonance Imaging Facility in Ithaca, New York or the York University Neuroimaging Center in Toronto, Canada^5^. MRI data from the Cornell University site was acquired on a 3T GE discovery MR750W 3T system with a 32-channel head coil. T1 weighted imaging was acquired with the following parameters: TR/TE = 2530/3.4 ms; 7° flip angle; 1 mm isotropic voxels, and 176 slices. MRI data from York University site was acquired on a 3T Siemens TimTrio MRI scanner with a 32-channel head coil. T1 weighted imaging was acquired from this site with the following parameters: TR/TE = 1900/2.52 ms, flip angle = 9°, 1 mm isotropic voxels, 192 slices.

*Paingen_placebo^6,7^*

Participants from the “Paingen_Placebo” data set were included in this analysis. MRI data was acquired on a Siemans Prisma 3T system with a 32-channel head coil at the University of Colorado at Boulder.^6^ T1 weighted imaging was acquired with the following parameters TR/TE = 2000/2.11 ms, flip angle = 8°, FOV = 256 mm, and resolution = 0.8 × 0.8 × 0.8 mm.

*AgeRisk^8,9^*

MRI data from the “AgeRisk” data set was acquired using a Siemens 3T MAGNETOM Prisma magnetic resonance imaging (MRI) system and a 20-channel head coil at the University Hospital Basel, Switzerland. T1-weighted MRI data was acquired using a magnetization-prepared rapid gradient echo sequence with the following parameters: TR/TE= 2500/4.25 ms, inversion time = 1100 ms, flip angle = 7^∘^, field of view = 256 mm × 256 mm, 192 slices, voxel dimensions = 1.0 mm isotropic.

**Table BI. Summary of Neurotypical Control Data Sets**

|  | NIMH^1,2^ | Neurocognitive Aging^3,4^ | Paingen-Placebo^5,6^ | Age-Risk^7,8^ |
| --- | --- | --- | --- | --- |
| n | 154 | 298 | 395 | 186 |
| Age (x̄ ± SD) | 34.0 ± 12.8 | 40.8 ± 23.0 | 36.1 ± 3.4 | 45.1 ± 19.3 |
| Age (min, max) | (18, 72) | (18, 89) | (27, 77) | (16, 81) |
| Males (n) | 53 | 131 | 163 | 89 |
| Females (n) | 101 | 166 | 232 | 97 |

**Figure B1. Violin Plot of Neurotypical Datasets Age.**

**Figure B2. Violin Plot of Neurotypical Datasets Compared with GM2 gangliosidosis patients.**

**Table B2. Summary of T1-Weighted Acquisition**

|  | GM2 Patients | NIMH^2,3^ | Neurocognitive Aging^4,5^ | Paingen-Placebo^6,7^ | Age-Risk^8,9^ |
| --- | --- | --- | --- | --- | --- |
| Brand | Phillips | GE | GE/Siemens | Siemens | Siemens |
| Coil Strength (T) | 3T | 3T | 3T | 3T | 3T |
| TR/TE (ms) | 8/4 | 6.95/2.92  7.35/3.04 | 2530/3.4  1900/2.52 | 2000/2.11 | 2500/4.25 |
| FOV (mm) | 220 |  | 131 | 256 | 256 x 256 |
| Voxel Dimensions (mm) | 1.0 x 1.0 x 1.0 | 1.0 x 1.0 x 1.0 \| 1.2 x 1.2 x 1.2 | 1.0 x 1.0 x 1.0 | 0.8 x 0.8 x 0.8 | 1.0 x 1.0 x 1.0 |
| Flip Angle (^∘^) | 8 | 8 \| 11 | 7 \| 9 | 8 | 7 |

**Supplementary Results**

**Supplement C: T1-Weighted Volumetric Analysis**

**Figure C1. Age related changes in gray matter volume.** LOSD patients are shown in red, adult LOTS patients are shown in blue, and neurotypical controls are shown as black points with shapes based on which Open-Source data set the scans were from. Total gray matter volume is shown in the top graph and gray matter volume as a percentage of intracranial volume (ICV) is shown in the bottom graph. No statistical differences were observed between the three cohorts for gray matter volume.

**Figure C2. Age related changes in white matter volume.** LOSD patients are shown in red, adult LOTS patients are shown in blue, and neurotypical controls are shown as black points with shapes based on which Open-Source data set the scans were from. Total white matter volume is shown in the top graph and white matter volume as a percentage of intracranial volume (ICV) is shown in the bottom graph. No statistical differences were observed between the three cohorts for white matter volume.

**Figure C3. Age related changes in thalamic volume.** LOSD patients are shown in red, LOTS patients are shown in blue, and neurotypical controls are shown as black points with shapes based on which Open-Source data set the scans were from. Total thalamic volume is shown in the top graph and thalamic volume as a percentage of intracranial volume (ICV) is shown in the bottom graph. No statistical differences were observed between the three cohorts for thalamic volume.

**Figure C4. Age related changes in ventricle volume.** LOSD patients are shown in red, LOTS patients are shown in blue, and neurotypical controls are shown as black points with shapes based on which Open-Source data set the scans were from. Total ventricle volume is shown in the top graph and thalamic volume as a percentage of intracranial volume (ICV) is shown in the bottom graph. No statistical differences were observed between the three cohorts for ventricle volume.

**Figure C5. Age related changes in caudate volume.** LOSD patients are shown in red, LOTS patients are shown in blue, and neurotypical controls are shown as black points with shapes based on which Open-Source data set the scans were from. Total caudate volume is shown in the top graph and caudate volume as a percentage of intracranial volume (ICV) is shown in the bottom graph. No statistical differences were observed between the three cohorts for ventricle volume.

**Figure C6. Age related changes in the volume of the 4^th^ ventricle.** LOSD patients are shown in red, adult LOTS patients are shown in blue, and neurotypical controls are shown as black points with shapes based on which Open-Source data set the scans were from. Total 4^th^ ventricle volume is shown in the top graph and 4^th^ ventricle volume as a percentage of intracranial volume (ICV) is shown in the bottom graph. Statistical differences were observed between GM2 patients and neurotypical controls (*p_corrected_* < 0.01), LOTS patients and neurotypical controls (*p_corrected_* < 0.01), and between LOTS and LOSD patients (*p_corrected_* = 0.04). However, there was no difference between LOSD patients and neurotypical controls (*p_corrected_* = 0.81)

**Figure C7. Age related changes in total intracranial volume.** LOSD patients are shown in red, LOTS patients are shown in blue, and neurotypical controls are shown as black points with shapes based on which Open-Source data set the scans were from. No statistical differences were observed between the three cohorts for total intracranial volume.

**Figure C8. Age related changes in ventricle volume.** LOSD patients are shown in red, LOTS patients are shown in blue, and neurotypical controls are shown as black points with shapes based on which Open-Source data set the scans were from. Total ventricle volume is shown in the top graph and thalamic volume as a percentage of intracranial volume (ICV) is shown in the bottom graph. No statistical differences were observed between the three cohorts for ventricle volume.

**Figure C9. Age related changes in left cerebellar gray matter volume.** LOSD patients are shown in red, LOTS patients are shown in blue, and neurotypical controls are shown as black points with shapes based on which Open-Source data set the scans were from. Total left cerebellar gray matter volume is shown in the top graph and left cerebellar gray matter volume as a percentage of intracranial volume (ICV) is shown in the bottom graph. Statistical differences were observed between GM2 patients and neurotypical controls (*p_corrected_* < 0.01), LOTS patients and neurotypical controls (*p_corrected_* < 0.01), and between LOTS patients and LOSD patients (*p_corrected_* < 0.01) for left cerebellar gray matter volume.

**Figure C10. Age related changes in left cerebellar white matter volume.** LOSD patients are shown in red, LOTS patients are shown in blue, and neurotypical controls are shown as black points with shapes based on which Open-Source data set the scans were from. Total left cerebellar white matter volume is shown in the top graph and left cerebellar white matter volume as a percentage of intracranial volume (ICV) is shown in the bottom graph. Statistical differences were observed between LOTS patients and neurotypical controls (*p_corrected_* < 0.01) and between LOTS patients and LOSD patients (*p_corrected_* < 0.01) for left cerebellar white matter volume.

**Figure C11. Age related changes in right cerebellar gray matter volume.** LOSD patients are shown in red, LOTS patients are shown in blue, and neurotypical controls are shown as black points with shapes based on which Open-Source data set the scans were from. Total left cerebellar gray matter volume is shown in the top graph and left cerebellar gray matter volume as a percentage of intracranial volume (ICV) is shown in the bottom graph. Statistical differences were observed between GM2 patients and neurotypical controls (*p_corrected_* < 0.01), LOTS patients and neurotypical controls (*p_corrected_* < 0.01), and between LOTS patients and LOSD patients (*p_corrected_* < 0.01) for left cerebellar gray matter volume.

**Figure C12. Age related changes in right cerebellar white matter volume.** ALOSD patients are shown in red, LOTS patients are shown in blue, and neurotypical controls are shown as black points with shapes based on which Open-Source data set the scans were from. Total left cerebellar white matter volume is shown in the top graph and left cerebellar white matter volume as a percentage of intracranial volume (ICV) is shown in the bottom graph. Statistical differences were observed between LOTS patients and neurotypical controls (*p_corrected_* < 0.01) and between LOTS patients and LOSD patients (*p_corrected_* < 0.01) for left cerebellar white matter volume.

**Supplement D: Diffusion Tensor Imaging Analysis Supplement Figures**

**
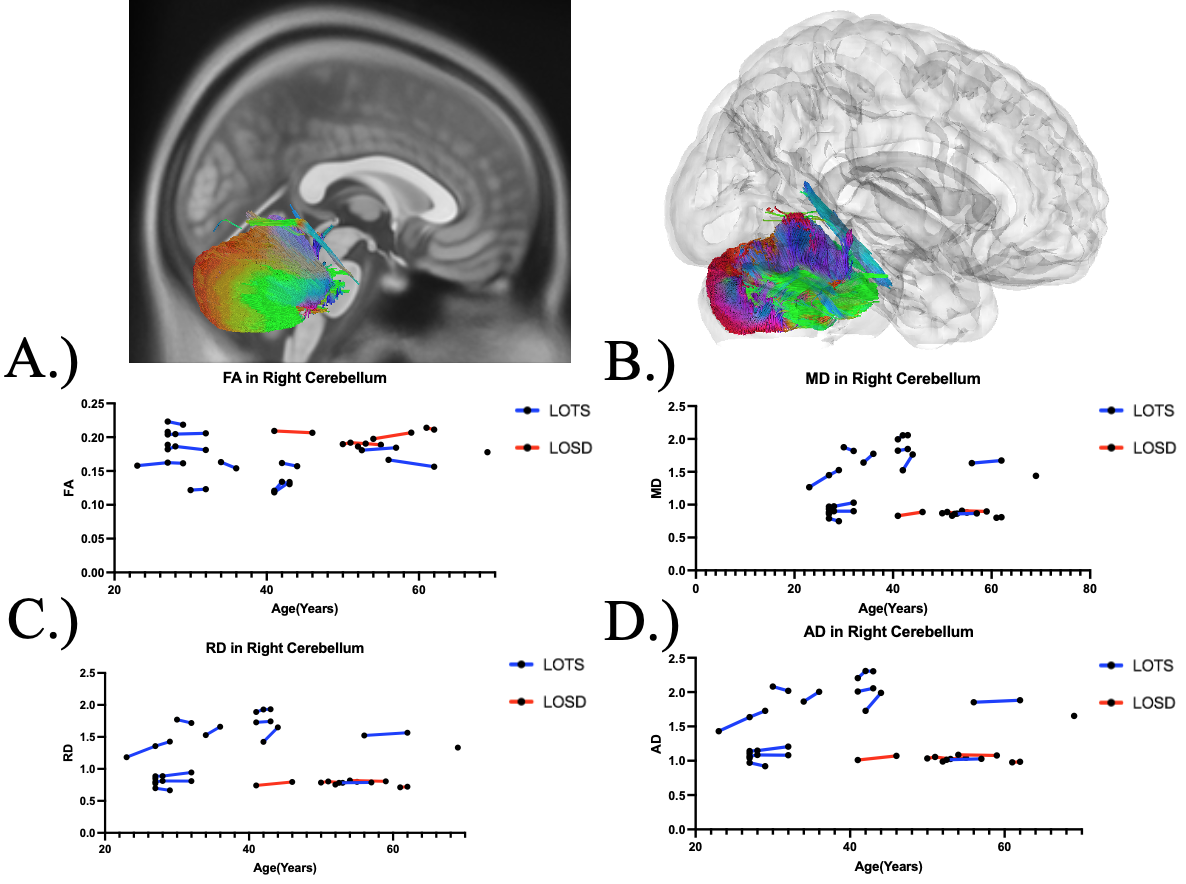
**

Figure D1. Atlas based fiber tractography of the right cerebellum demonstrating age related effects on A.) fractional anisotropy B.) mean diffusivity between C.) radial diffusivity D.) axial diffusivity between LOTS patients (blue) and LOSD patients (red). LOTS patients demonstrated decreased FA (𝜒^2^(1) = 5.50, *p =* 0.02) and increased MD (𝜒^2^(1) = 8.56, *p <* 0.01), RD (𝜒^2^(1) = 8.55, *p <* 0.01), and AD (𝜒^2^(1) = 8.57, *p <* 0.01) compared to LOSD patients in fiber tracts in the right cerebellum when age was accounted for.


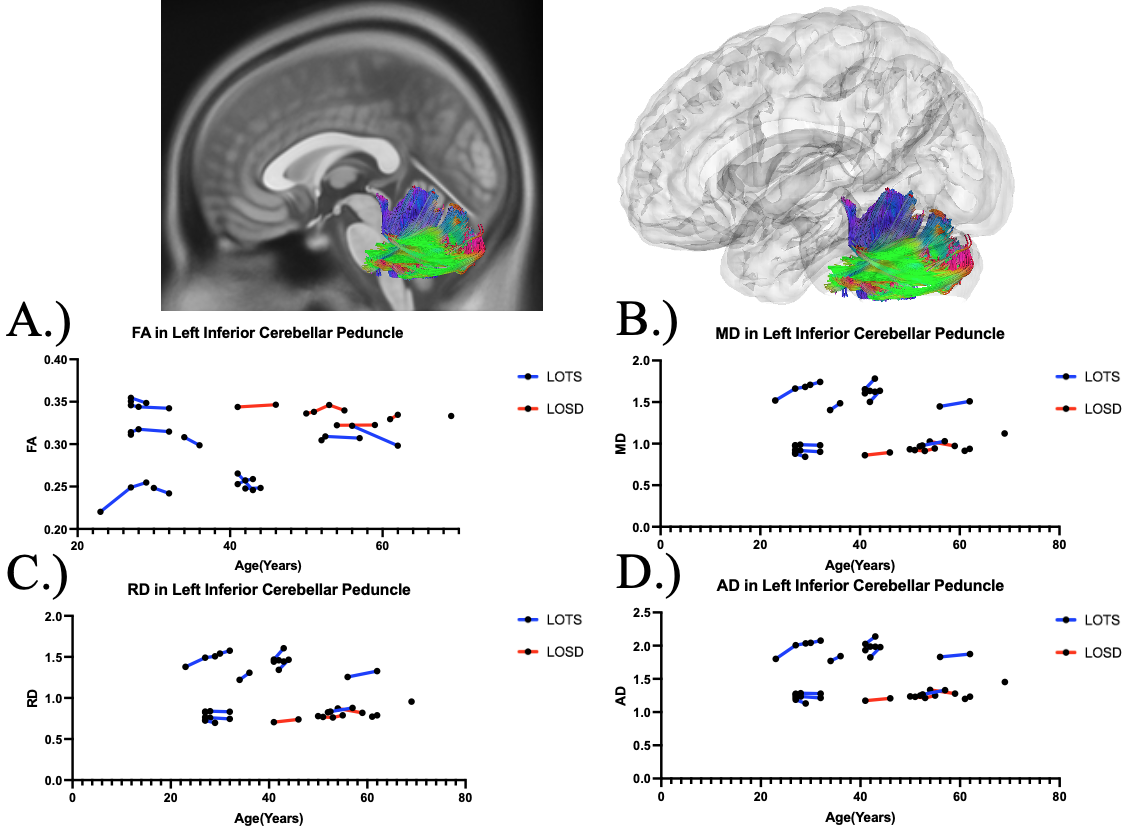


Fig D2. Atlas based fiber tractography of the left inferior cerebellar peduncle demonstrating age related effects on A.) fractional anisotropy B.) mean diffusivity between C.) radial diffusivity D.) axial diffusivity between LOTS patients (blue) and LOSD patients (red). LOTS patients demonstrated no difference in FA (𝜒^2^(1) = 3.10, *p =* 0.08) and increased MD (𝜒^2^(1) = 7.02, *p <* 0.01), RD (𝜒^2^(1) = 6.85, *p <* 0.01), and AD (𝜒^2^(1) = 7.26, *p <* 0.01) compared to LOSD patients in fiber tracts in the left inferior cerebellar peduncle when age was accounted for.

**
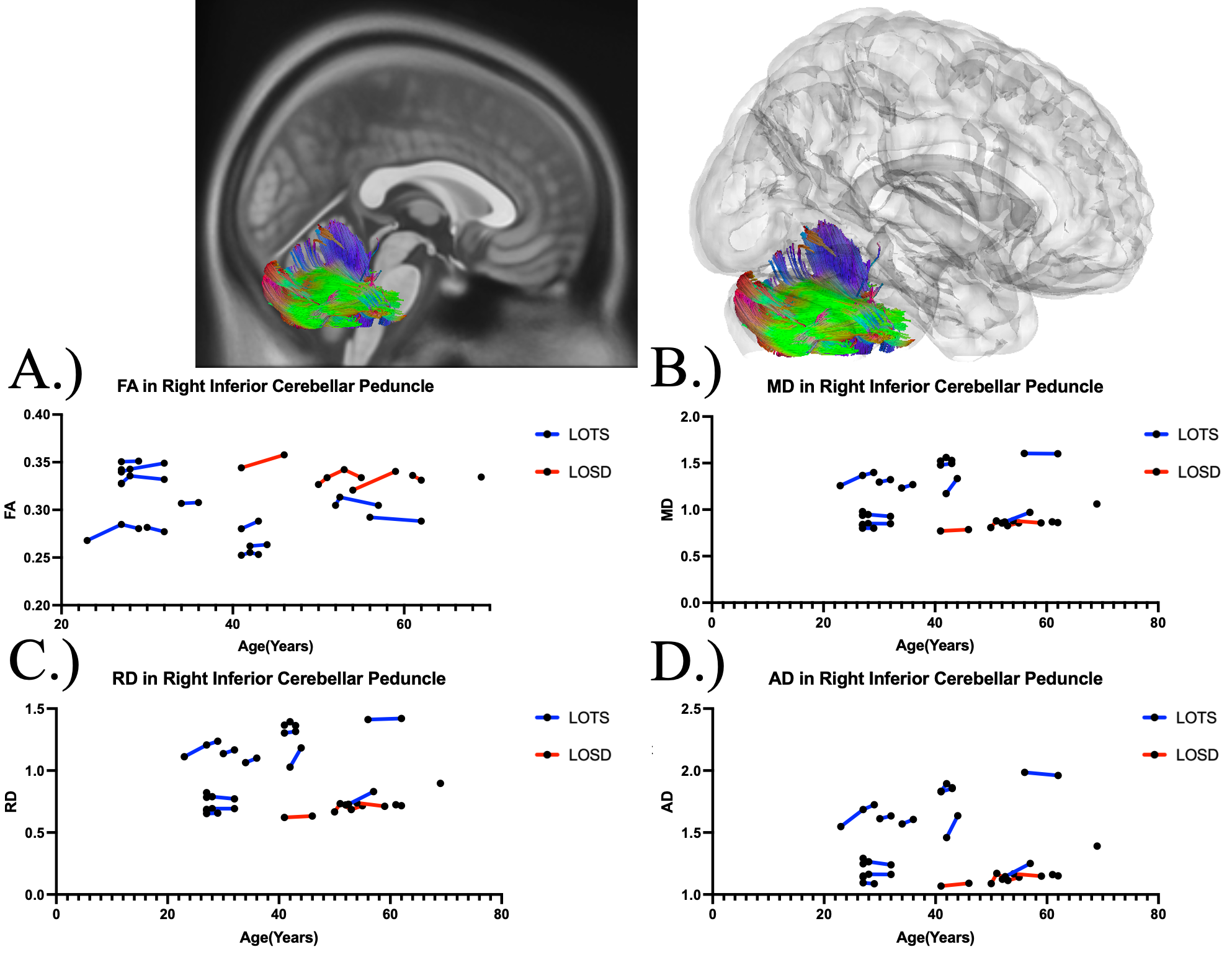
**

Fig D3. Atlas Based Fiber Tractography of the right inferior cerebellar peduncle demonstrating age related effects on A.) fractional anisotropy B.) mean diffusivity between LOTS patients (blue) and LOSD patients (red). LOTS patients demonstrated no difference in FA (𝜒^2^(1) = 1.89, *p =* 0.17) and increased MD (𝜒^2^(1) = 8.55, *p <* 0.01), RD (𝜒^2^(1) = 8.39, *p <* 0.01), and AD (𝜒^2^(1) = 8.79, *p <* 0.01) compared to LOSD patients in fiber tracts in the right inferior cerebellar peduncle when age was accounted for.


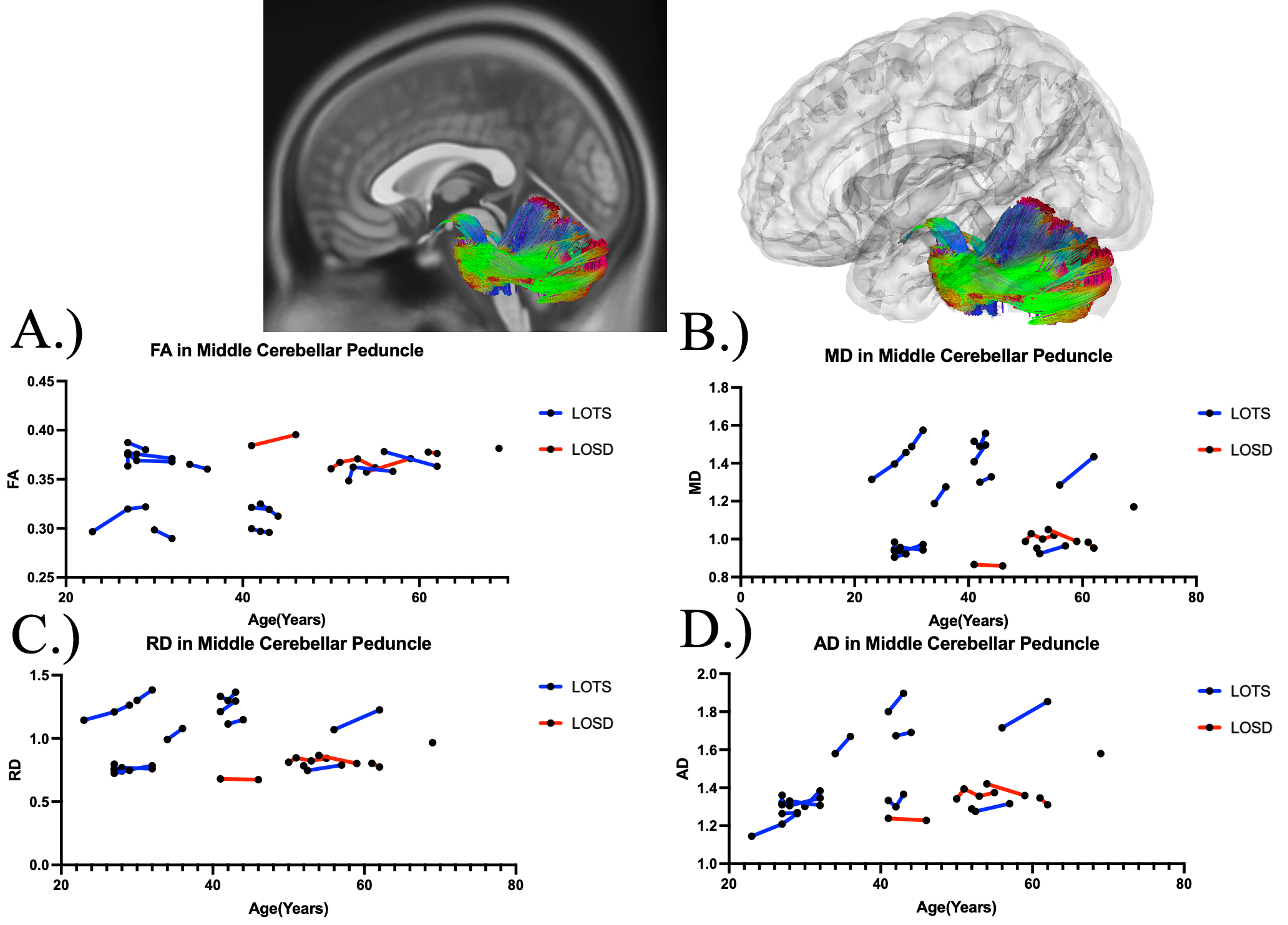


Fig D4. Atlas Based Fiber Tractography of the middle cerebellar peduncle demonstrating age related effects on A.) fractional anisotropy B.) mean diffusivity between LOTS patients (blue) and LOSD patients (red). LOTS patients demonstrated no difference in FA (𝜒^2^(1) = 1.65, *p =* 0.20) and increased MD (𝜒^2^(1) = 6.81, *p <* 0.01), RD (𝜒^2^(1) = 6.55, *p =* 0.01), and AD (𝜒^2^(1) = 7.22, *p <* 0.01) compared to LOSD patients in fiber tracts in the middle cerebellar peduncle when age was accounted for.


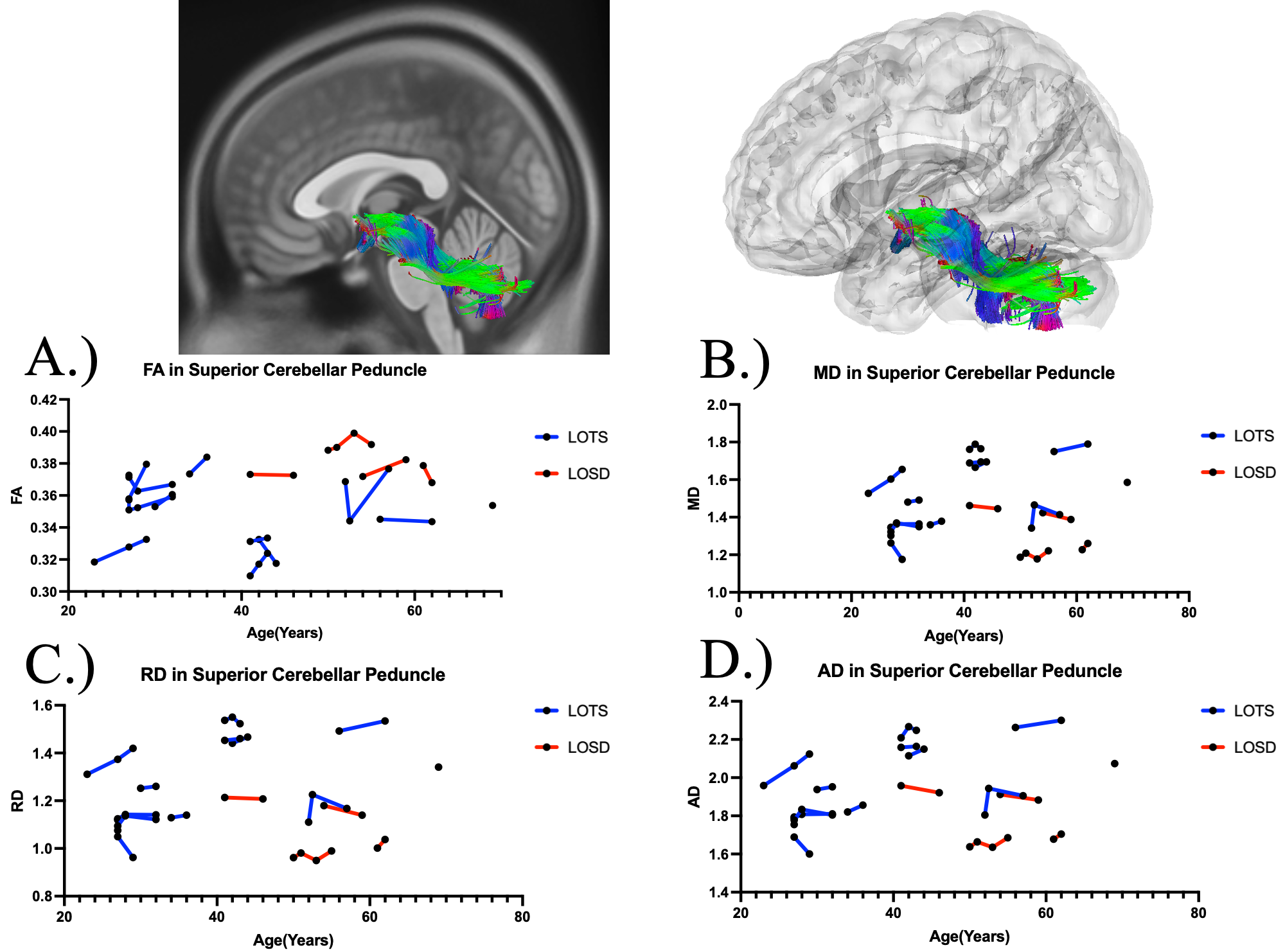


Fig D5. Atlas Based Fiber Tractography of the superior cerebellar peduncle (SCP) demonstrating age related effects on A.) fractional anisotropy B.) mean diffusivity between LOTS patients (blue) and LOSD patients (red). LOTS patients demonstrated lower FA (𝜒^2^(1) = 4.80, *p =* 0.03) and increased MD (𝜒^2^(1) = 7.19, *p <* 0.01), RD (𝜒^2^(1) = 7.18, *p <* 0.01), and AD (𝜒^2^(1) = 7.09, *p <* 0.01) compared to LOSD patients in fiber tracts in the SCP when age was accounted for.


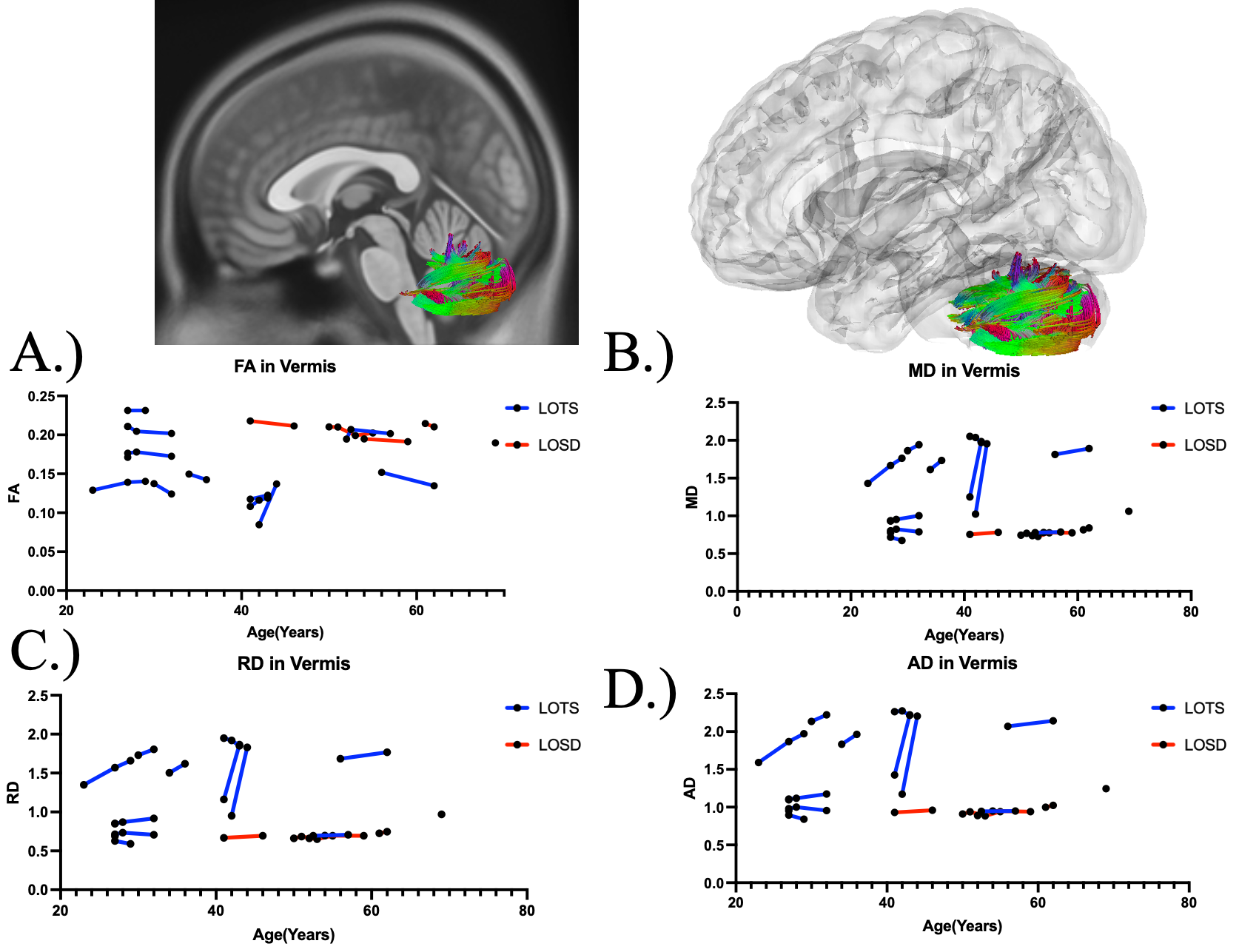


Fig D6. Atlas Based Fiber Tractography of the cerebellar vermis demonstrating age related effects on A.) fractional anisotropy B.) mean diffusivity between LOTS patients (blue) and LOSD patients (red). LOTS patients demonstrated lower FA (𝜒^2^(1) = 5.05, *p =* 0.03) and increased MD (𝜒^2^(1) = 6.62, *p =* 0.01), RD (𝜒^2^(1) = 6.70, *p <* 0.01), and AD (𝜒^2^(1) = 6.47, *p =* 0.01) compared to LOSD patients in fiber tracts in the cerebellar vermis when age was accounted for.


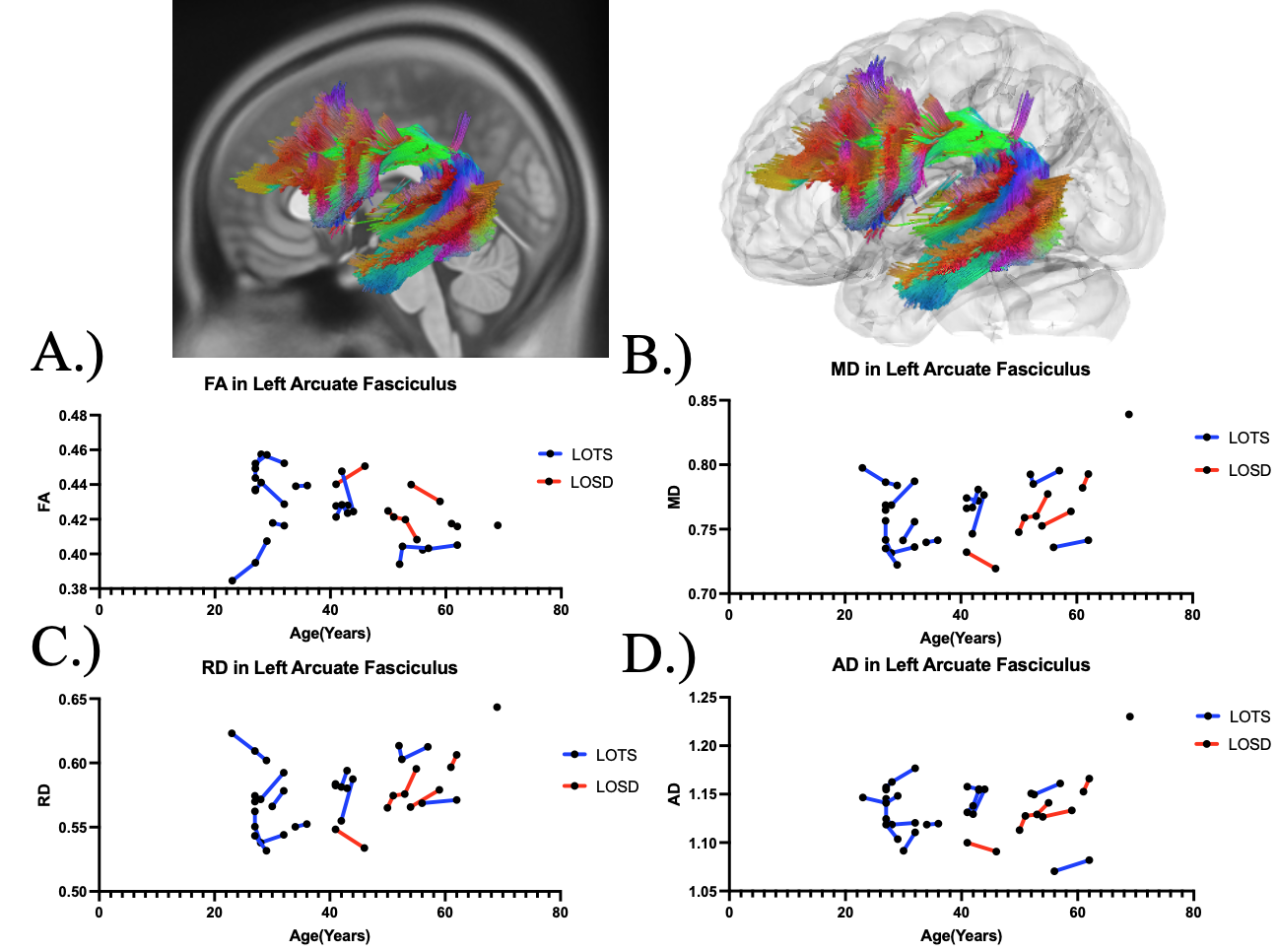


Fig D7. Atlas Based Fiber Tractography of the left arcuate fasciculus demonstrating age related effects on A.) fractional anisotropy B.) mean diffusivity between LOTS patients (blue) and LOSD patients (red). There was no statistical difference between LOTS and LOSD patients in FA (𝜒^2^(1) = 0.92, *p =* 0.34), MD (𝜒^2^(1) = 2.65, *p =* 0.10), RD (𝜒^2^(1) = 2.66, *p =* 0.10), or AD (𝜒^2^(1) = 2.02, *p =* 0.16) in fiber tracts in the left arcuate fasciculus.


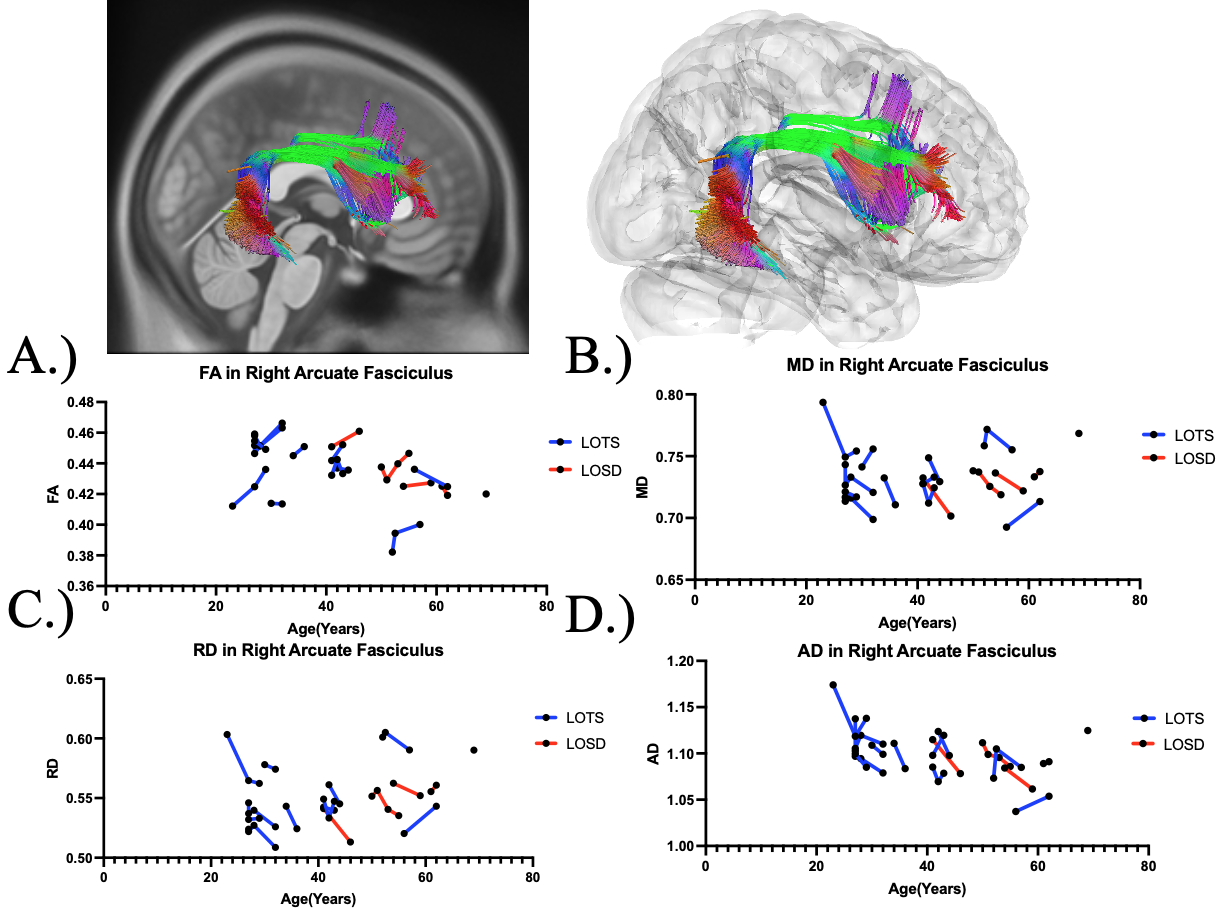


Fig D8. Atlas Based Fiber Tractography of the right arcuate fasciculus demonstrating age related effects on A.) fractional anisotropy B.) mean diffusivity between LOTS patients (blue) and LOSD patients (red). There was no statistical difference between LOTS and LOSD patients in FA (𝜒^2^(1) = 0.04, *p =* 0.83), MD (𝜒^2^(1) = 0.12, *p =* 0.73), RD (𝜒^2^(1) = 0.14, *p =* 0.71), or AD (𝜒^2^(1) = 0.08, *p =* 0.78) in fiber tracts in the right arcuate fasciculus.


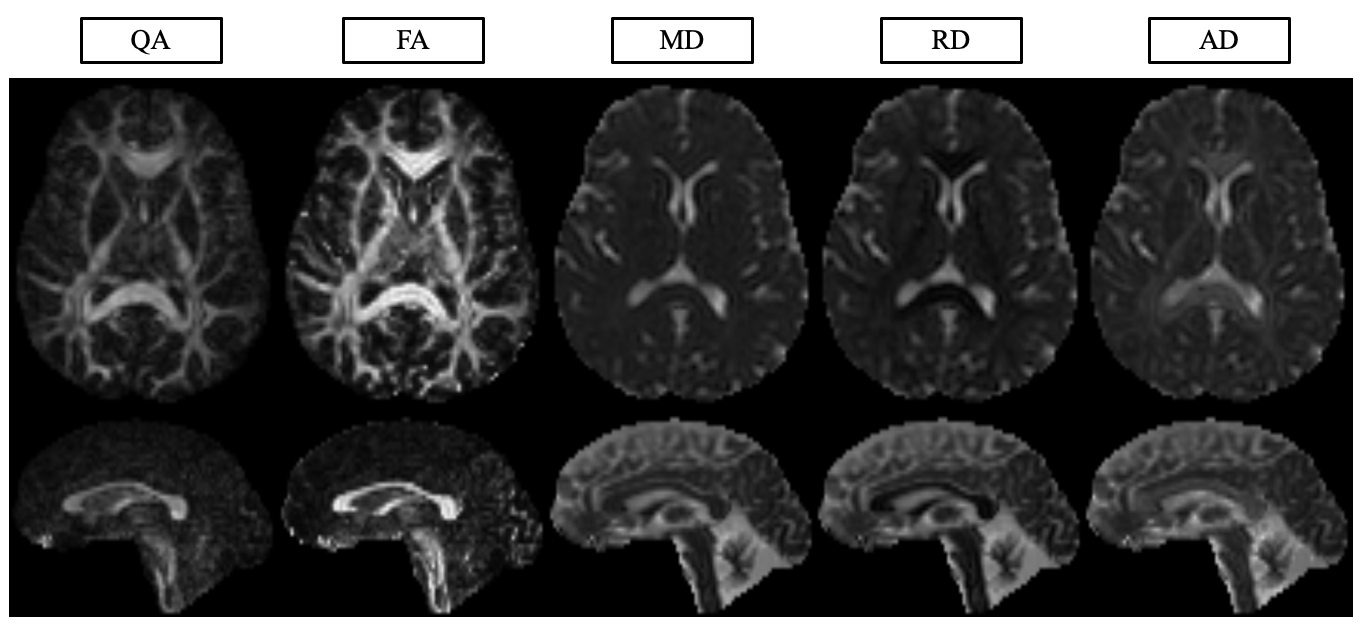


Figure D9. Quantitative Anisotropy (QA), Fractional Anisotropy (FA), Mean Diffusivity (MD), Radial Diffusivity (RD), and Axial Diffusivity (AD) imaging.

**Supplement E. Estimates and Standard Error of Volumetric Linear Mixed Effects Modeling (LMEM).**

**Table EI. T1-Weighted Volumetric MRI Analysis Estimates and Standard Errors of LMEM. all volumes were controlled ICV. *P*-values < 0.01 were considered significant and bolded.**

| Structure | *GM2 v NC* | *LOSD v NC* | *LOTS v NC* | *LOSD v LOTS* |
| --- | --- | --- | --- | --- |
| Estimate  (Standard Error) |  |  |  |  |
| Gray Matter Volume | 0.0006059  (0.0159794) | 0.0118404  (0.0314622) | 0.0039526 (0.0185003) | 0.0412318 (0.0278134) |
| White Matter Volume | 0.0077523  (0.0131926) | 0.0276347  (0.0260632) | 0.0005996 (0.0152424) | 0.0409341 (0.0188115) |
| Cerebellum | **-0.01978**  (0.004161) | 0.003808  (0.007993) | -0.02826  (0.004807) | **0.0432069 (0.0123726)** |
| Left Cerebellar White Matter | **-0.001171**  (0.0005406) | 0.001548  (0.001042) | -0.002153 (0.0006252) | 0.005476 (0.001398) |
| Right Cerebellar White Matter | **-0.001143**  (0.0005293) | 0.001421  (0.001017) | -0.002072 (0.0006130) | 0.004210 (0.001269) |
| Left Cerebellar Gray Matter | **-0.008184**  (0.001637) | -0.0002429  (0.003294) | -0.01153 0.001894 | 0.0163413 (0.0051302) |
| Right Cerebellar Gray Matter | **-0.009253**  (0.001702) | -0.001094  (0.003128) | -0.01249 (0.001964) | 0.0166357 (0.0050131) |
| Ventricles | -0.001380  (0.001847) | -0.0004512  (0.003612) | -0.001727 (0.002143) | 0.004361 (0.003011) |
| 4^th^ Ventricle | **0.0004701**  (0.0001039) | -0.0002615  0.0002054 | 0.0007340  (0.0001178) | -0.0007746 (0.0002969) |
| Thalamus | -0.0007720  0.0004481 | -0.0001468  (0.0008863) | -0.0009981 (0.0005175) | 0.001468 (0.0005818) |
| Caudate | 0.000456  (0.0002332) | 0.0003022  (0.0004596) | 0.0005120 (0.0002692) | 0.0002090 (0.0004979) |
| Intracranial Volume (ICV) | -140000  (57126.5) | -127907.6  (112809.5) | -144131.0 (66089.9) | -56296  (78536) |
| Brainstem | -0.0007558  (0.0007124) | -0.0004370  (0.001401) | -0.0008735 (0.0008261) | 0.001400 (0.0008710) |

**Supplement F. Diffusion Tensor Imaging Analysis of RD, AD, and QA from Linear Mixed Effects Modeling (LMEM).**

**Table F1. Diffusion Tensor Imaging Results of RD in Atlas Fiber Tractography Pathways evaluating differences between LOTS and LOSD Patients.**

| Pathway | Estimate | Standard Error | 𝜒^2^(1) | *p-value* (>𝜒^2^) |
| --- | --- | --- | --- | --- |
| Whole Brain | -0.09 | 0.03 | 7.28 | **< 0.01** |
| Left Cerebellum | -0.71 | 0.23 | 8.51 | **< 0.01** |
| Right Cerebellum | -0.70 | 0.23 | 8.55 | **< 0.01** |
| Left Inferior Cerebellar Peduncle | -0.48 | 0.18 | 6.85 | **< 0.01** |
| Right Inferior Cerebellar Peduncle | -0.42 | 0.14 | 8.39 | **< 0.01** |
| Middle Cerebellar Peduncle | -0.33 | 0.13 | 6.55 | **0.01** |
| Superior Cerebellar Peduncle | -0.26 | 0.09 | 7.18 | **< 0.01** |
| Vermis | -0.70 | 0.27 | 6.70 | **< 0.01** |
| Corpus Callosum | < 0.01 | 0.02 | 0.02 | 0.90 |
| Left Arcuate Fasciculus | -0.02 | 0.01 | 2.66 | 0.10 |
| Right Arcuate Fasciculus | < -0.01 | 0.02 | 0.14 | 0.71 |

**Table F2. Diffusion Tensor Imaging Results of AD in Atlas Fiber Tractography Pathways evaluating differences between LOTS and LOSD Patients.**

| Pathway | Estimate | Standard Error | 𝜒^2^(1) | *p-value* (>𝜒^2^) |
| --- | --- | --- | --- | --- |
| Whole Brain | -0.10 | 0.03 | 7.86 | **< 0.01** |
| Left Cerebellum | -0.76 | 0.25 | 8.25 | **< 0.01** |
| Right Cerebellum | -0.75 | 0.24 | 8.57 | **< 0.01** |
| Left Inferior Cerebellar Peduncle | -0.55 | 0.20 | 7.26 | **< 0.01** |
| Right Inferior Cerebellar Peduncle | -0.48 | 0.16 | 8.79 | **< 0.01** |
| Middle Cerebellar Peduncle | -0.37 | 0.14 | 7.22 | **< 0.01** |
| Superior Cerebellar Peduncle | -0.28 | 0.10 | 7.09 | **< 0.01** |
| Vermis | -0.75 | 0.30 | 6.47 | **0.01** |
| Corpus Callosum | < 0.01 | 0.03 | 0.01 | 0.91 |
| Left Arcuate Fasciculus | -0.03 | 0.02 | 2.02 | 0.16 |
| Right Arcuate Fasciculus | < 0.01 | 0.01 | 0.08 | 0.78 |

**Table F3. Diffusion Tensor Imaging Results of QA in Atlas Fiber Tractography Pathways evaluating differences between LOTS and LOSD Patients.**

| Pathway | Estimate | Standard Error | 𝜒^2^(1) | *p-value* (>𝜒^2^) |
| --- | --- | --- | --- | --- |
| Whole Brain | < -0.01 | 0.01 | 0.68 | 0.41 |
| Left Cerebellum | 0.03 | 0.02 | 3.57 | 0.06 |
| Right Cerebellum | 0.02 | 0.02 | 1.53 | 0.22 |
| Left Inferior Cerebellar Peduncle | 0.02 | 0.02 | 1.15 | 0.28 |
| Right Inferior Cerebellar Peduncle | < 0.01 | 0.02 | 0.02 | 0.90 |
| Middle Cerebellar Peduncle | 0.02 | 0.02 | 1.47 | 0.22 |
| Superior Cerebellar Peduncle | < 0.01 | 0.02 | 0.16 | 0.69 |
| Vermis | 0.03 | 0.02 | 2.89 | 0.09 |
| Corpus Callosum | -0.02 | 0.01 | 2.65 | 0.10 |
| Left Arcuate Fasciculus | -0.01 | 0.01 | 0.73 | 0.39 |
| Right Arcuate Fasciculus | -0.02 | 0.01 | 2.39 | 0.12 |

**Supplement G: Correlational Fiber Tractography Analysis, Supplement Figures**


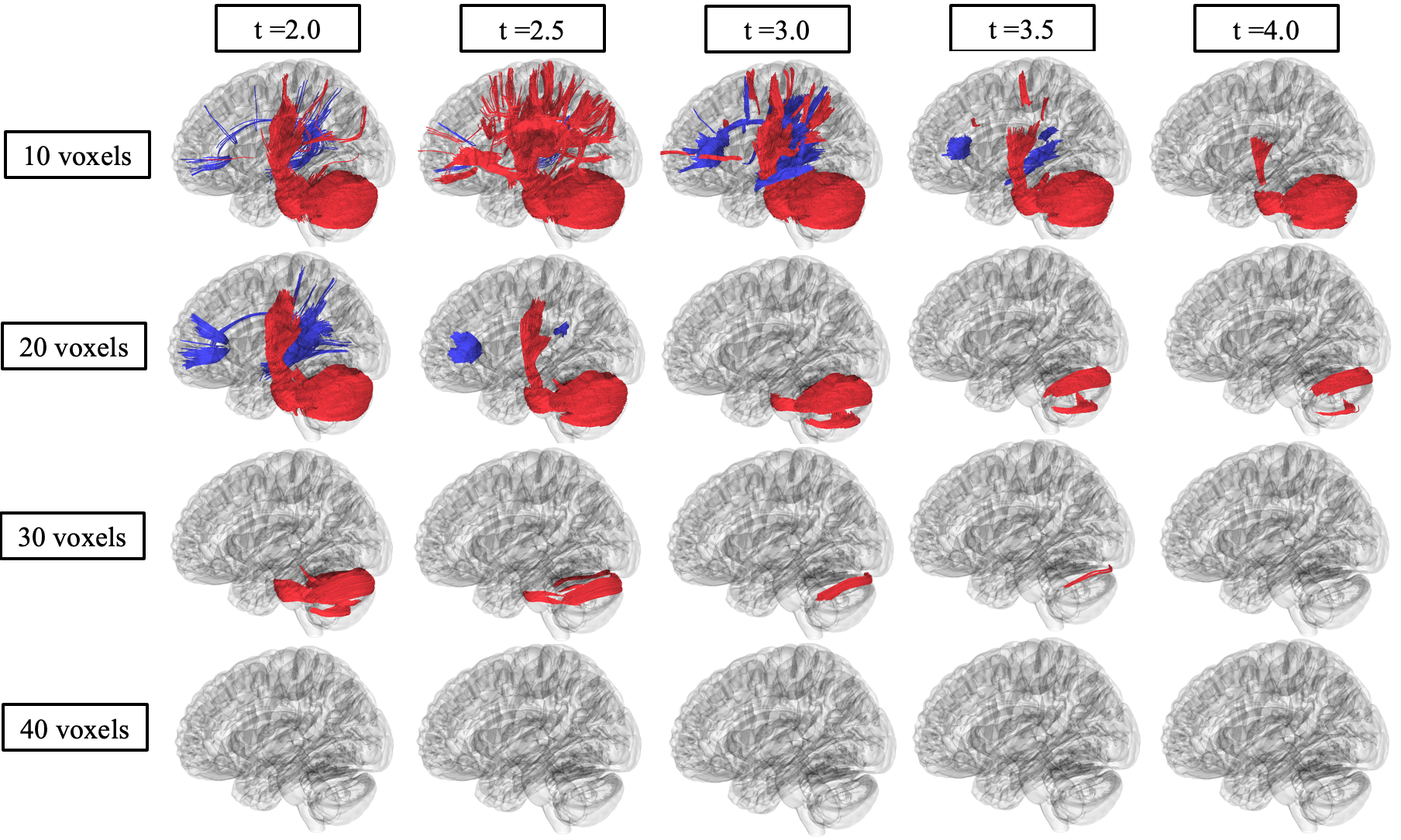


Figure G1. Correlational fiber tractography assessed differences in quantitative anisotropy (QA) in LOSD and LOTS patients at varying length (voxels) and T thresholds. Fiber tracts shown in red were evaluated to have a higher quantitative anisotropy in LOSD patients compared to LOTS patients and were observed primarily in the cerebellum (FDR < 0.05). Fiber tracts shown in blue were evaluated to have a higher quantitative anisotropy in LOTS patients compared to LOSD patients.


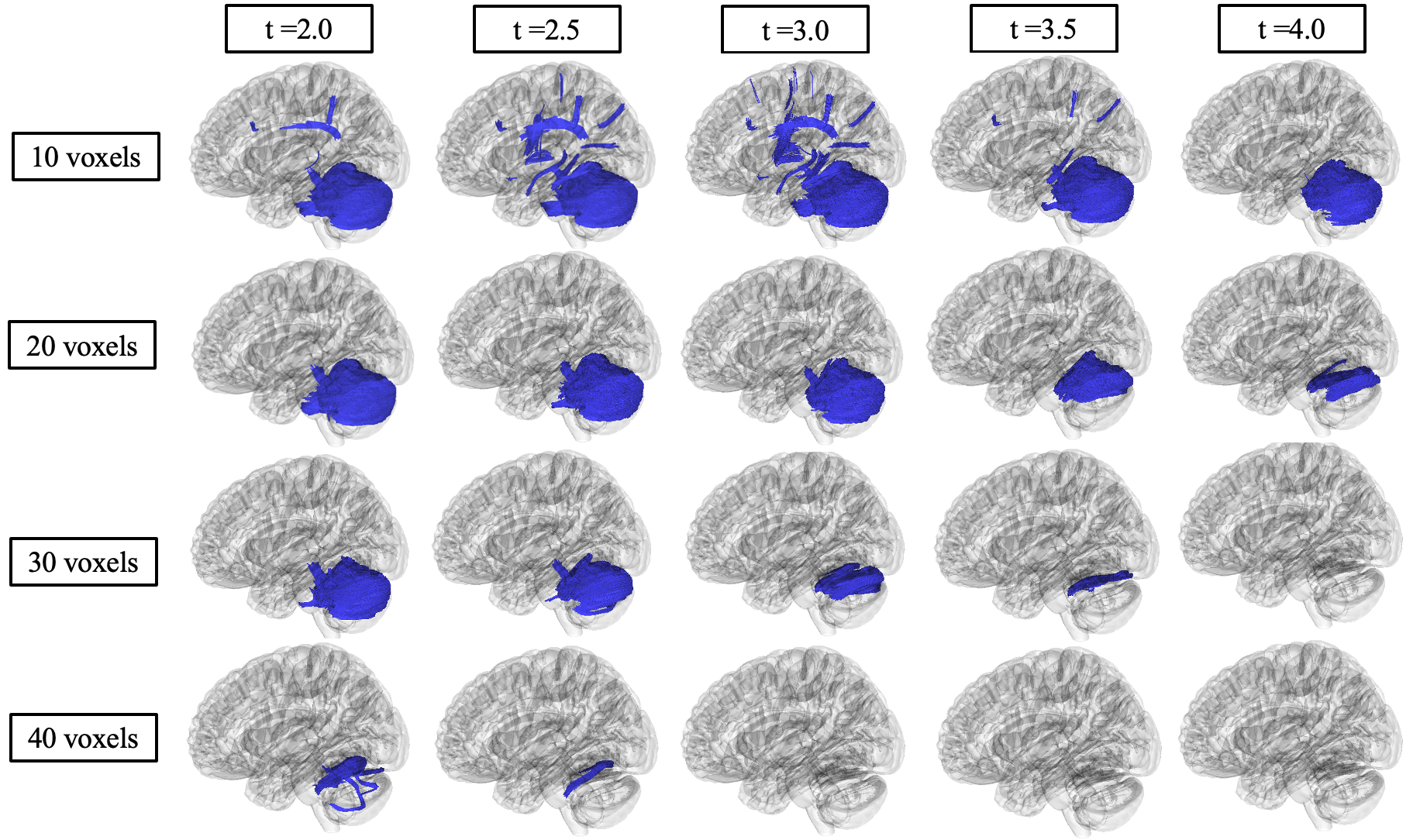


Figure G2. Correlational fiber tractography assessed differences in radial diffusivity (RD) in LOSD and LOTS patients at varying length (voxels) and T thresholds. Fiber tracts shown in blue were evaluated to have a higher radial diffusivity in LOTS patients compared to LOSD patients and were observed primarily in the cerebellum (FDR < 0.05). No fiber tracts were evaluated to have a higher radial diffusivity in LOSD patients compared to LOTS patients (red).


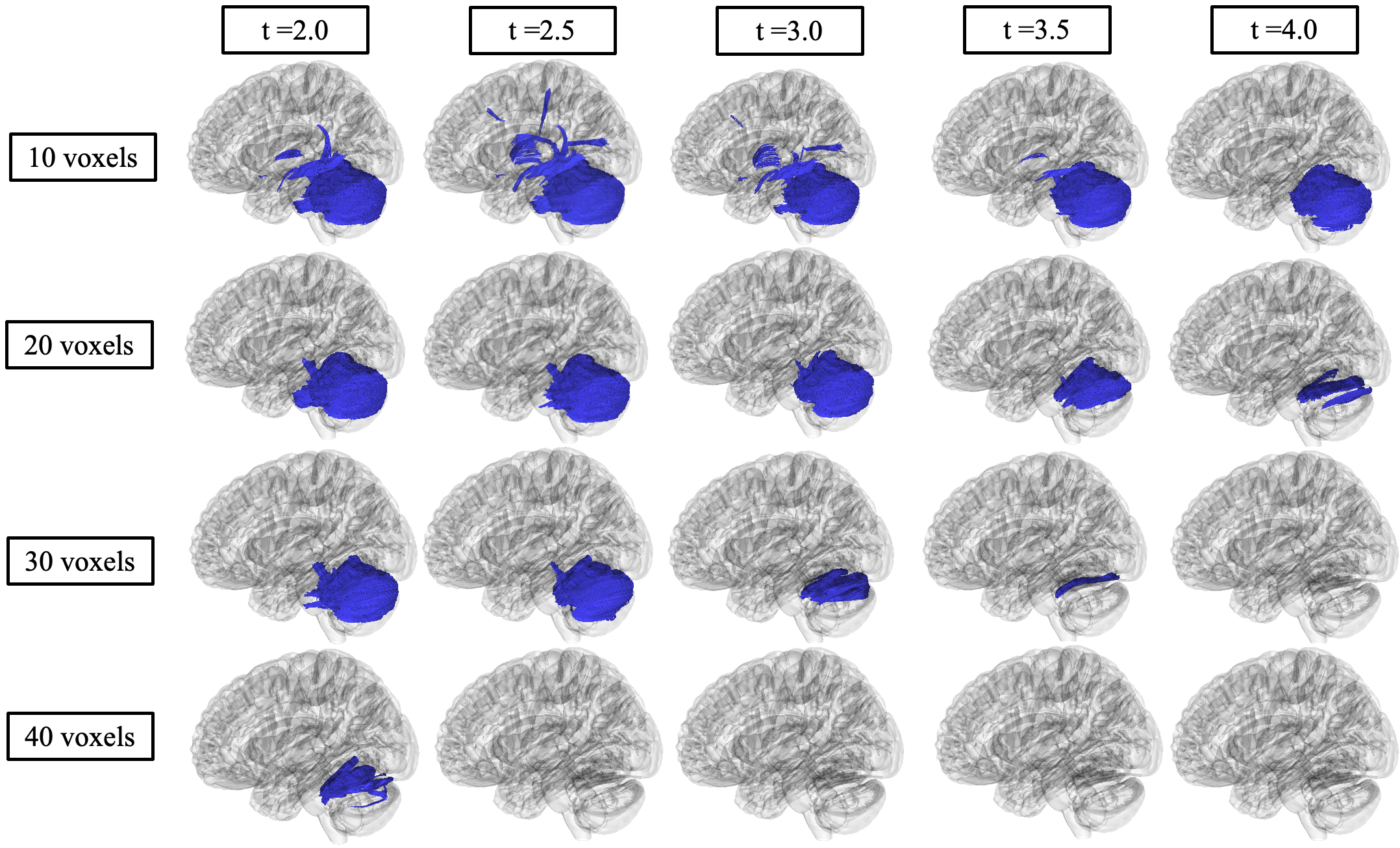


Figure G3. Correlational fiber tractography assessed differences in axial diffusivity (AD) in LOSD and LOTS patients at varying length (voxels) and T thresholds. Fiber tracts shown in blue were evaluated to have a higher axial diffusivity in LOTS patients compared to LOSD patients and were observed primarily in the cerebellum (FDR < 0.05). No fiber tracts were evaluated to have a higher axial diffusivity in LOSD patients compared to LOTS patients (red).

**Supplementary References**

1. National Human Genome Research Institute. Natural History of Glycosphingolipid Storage Disorders and Glycoprotein Disorders ClinicalTrials.gov identifier: NCT00029965. Updated August 7, 2024. Accessed August 12, 2024. <https://clinicaltrials.gov/study/NCT00029965>.
2. Nugent, A. C., Thomas, A. G., Mahoney, M., Gibbons, A., Smith, J. T., Charles, A. J., Shaw, J. S., Stout, J. D., Namyst, A. M., Basavaraj, A., Earl, E., Riddle, T., Snow, J., Japee, S., Pavletic, A. J., Sinclair, S., Roopchansingh, V., Bandettini, P. A., & Chung, J. (2022). The NIMH intramural healthy volunteer dataset: A comprehensive MEG, MRI, and behavioral resource. Scientific Data, 9, Article 518. https://doi.org/10.1038/s41597-022-01623-9
3. Allison C. Nugent and Adam G Thomas and Margaret Mahoney and Alison Gibbons and Jarrod Smith and Antoinette Charles and Jacob S Shaw and Jeffrey D Stout and Anna M Namyst and Arshitha Basavaraj and Eric Earl and Travis Riddle and Joseph Snow and Shruti Japee and Adriana Pavletic and Stephen Sinclair and Vinai Roopchansingh and Peter A Bandettini and Joyce Chung (2023). The NIMH Healthy Research Volunteer Dataset. OpenNeuro. [Dataset] doi: doi:10.18112/openneuro.ds004215.v1.0.3
4. Spreng, R.N., Setton, R., Alter, U. *et al.* Neurocognitive aging data release with behavioral, structural and multi-echo functional MRI measures. *Sci Data* **9**, 119 (2022). <https://doi.org/10.1038/s41597-022-01231-7>
5. R. Nathan Spreng and Roni Setton and Udi Alter and Benjamin N. Cassidy and Bri Darboh and Elizabeth DuPre and Karin Kantarovich and Amber W. Lockrow and Laetitia Mwilambwe-Tshilobo and Wen-Ming Luh and Prantik Kundu and Gary R. Turner (2022). Neurocognitive aging data release with behavioral, structural, and multi-echo functional MRI measures. OpenNeuro. [Dataset] doi: doi:10.18112/openneuro.ds003592.v1.0.13
6. Botvinik-Nezer, R., Petre, B., Ceko, M. *et al.* Placebo treatment affects brain systems related to affective and cognitive processes, but not nociceptive pain. *Nat Commun* **15**, 6017 (2024). <https://doi.org/10.1038/s41467-024-50103-8>
7. Rotem Botvinik-Nezer and Bogdan Petre and Marta Ceko and Naomi Friedman and Tor Wager (2023). Paingen_placebo. OpenNeuro. [Dataset] doi: doi:10.18112/openneuro.ds004746.v1.0.1
8. Tisdall, L., Mata, R. Age differences in the neural basis of decision-making under uncertainty. Cogn Affect Behav Neurosci 23, 788–808 (2023). https://doi.org/10.3758/s13415-022-01060-6
9. Tisdall, Loreen and Mata, Rui (2023). AgeRisk. OpenNeuro. [Dataset] doi: doi:10.18112/openneuro.ds004711.v1.0.0
